# CS-count-optimal quantum circuits for arbitrary multi-qubit unitaries

**DOI:** 10.1038/s41598-024-64558-8

**Published:** 2024-06-17

**Authors:** Priyanka Mukhopadhyay

**Affiliations:** Department of Computer Science, University of Toronto, Toronto, ON Canada

**Keywords:** Quantum information, Computer science

## Abstract

In quantum computing there are quite a few universal gate sets, each having their own characteristics. In this paper we study the Clifford+CS universal fault-tolerant gate set. The CS gate is used is many applications and this gate set is an important alternative to Clifford+T. We introduce a generating set in order to represent any unitary implementable by this gate set and with this we derive a bound on the CS-count of arbitrary multi-qubit unitaries. Analysing the channel representation of the generating set elements, we infer ${\mathcal {J}}_n^{CS}\subset {\mathcal {J}}_n^T$, where ${\mathcal {J}}_n^{CS}$ and ${\mathcal {J}}_n^T$ are the set of unitaries exactly implementable by the Clifford+CS and Clifford+T gate sets, respectively. We develop CS-count optimal synthesis algorithms for both approximately and exactly implementable multi-qubit unitaries. With the help of these we derive a CS-count-optimal circuit for Toffoli, implying ${\mathcal {J}}_n^{Tof}={\mathcal {J}}_n^{CS}$, where ${\mathcal {J}}_n^{Tof}$ is the set of unitaries exactly implementable by the Clifford+Toffoli gate set. Such conclusions can have an important impact on resource estimates of quantum algorithms.

## Introduction

One of the popular models for describing and implementing quantum algorithms is quantum circuits, which consist of a series of elementary operations or gates belonging to a universal set. This set usually consists of Clifford group gates and at least one non-Clifford gate^1,2^. Few examples of universal gate sets are Clifford+T, V-basis, Clifford+CS, Toffoli+H. The choice of which universal gate set to use depends on a number of factors, for example the underlying hardware technology. Irrespective of this choice, an important component of any quantum computer compilation process is quantum circuit synthesis and optimization.

Just like its classical counterpart, controlling the error in hardware components like gates, measurement devices, etc is an important challenge. These errors, if allowed to accumulate can become large enough to render the whole computation unreliable. Using quantum error correction and fault-tolerant design is one of the ways for countering this caveat. But this itself comes with its own challenges, which arises due to the use of extra qubits, gates and other gadgets, that themselves maybe error-prone. Thus an important part of quantum circuit compilation is the optimization of these error-prone components.

If we are working in the NISQ era, then optimization of multi-qubit gates like CNOT is more relevant because of their lower fidelity compared to single-qubit gates. While working in the fault-tolerant regime, optimization of non-Clifford gates are more relevant because of their higher overhead compared to the non-Clifford ones^3,4^. Thus the optimization of multi-qubit non-Clifford gate like CS is important for both the NISQ as well as fault-tolerant regime. It is also worth noting that the minimum number of non-Clifford gates required to implement certain unitaries is a quantifier of difficulty in many algorithms that try to classically simulate qunatum computation^5,6^.

According to the Solovay-Kitaev algorithm^7,8^ we can generate a circuit for an *n*-qubit unitary *W* with a “discrete finite” universal gate set, like Clifford+T, V-basis, Clifford+CS, Clifford+Toffoli. The unitary *U* implemented by the circuit is at most a certain distance from *W*. In fact, in quantum computation a set of gates is said to be universal if any quantum operation can be approximated to arbitrary accuracy by a quantum circuit involving only these gates^2^. A unitary is called exactly implementable by a gate set if there exists a quantum circuit with these gates, that implements it (up to some global phase). Otherwise, it is approximately implementable. Accordingly, a synthesis algorithm can be (a) exact when $U=e^{i\phi }W$ ($\phi$ is the global phase); or (b) approximate when $d(U,W)\le \epsilon$ for some $\epsilon >0$. *d*(.) is a distance metric and for quantum circuit synthesis two popular metrics are the operator norm^9,10^ and global phase invariant distance^11–15^. In this paper we use the global phase invariant distance because it ignores the global phase and hence avoids unnecessarily long approximating sequences that achieves a specific global phase. This distance is composable and inequalities relating it to operator norm can be found in^14^. It has also been used to synthesize unitaries in other models like topological quantum computation^16,17^.

In this paper we consider the Clifford+CS gate set, where the CS gate is defined as follows:

1 $$\begin{aligned} CS=\begin{bmatrix} 1 &{} 0 &{} 0 &{} 0 \\ 0 &{} 1 &{} 0 &{} 0 \\ 0 &{} 0 &{} 1 &{} 0 \\ 0 &{} 0 &{} 0 &{} i \\ \end{bmatrix} \end{aligned}$$

This gate set is universal for quantum computing and a fault-tolerant implementation via magic state distillation has been shown in^18,19^. Due to its natural implementation as an entangling operation in certain superconducting qubit systems whose fidelity is approaching that of single-qubit gates^20–25^, it has received much attention as a non-Clifford alternative to the T gate. Algorithms have been developed to synthesize CS-count optimal circuit for 2-qubit exactly implementable unitaries^26^.

### Our contributions

In this section we discuss briefly our motivations for studying some problems related to this gate set. We also enumerate and discuss the main results in our paper.

We already mentioned the importance of optimizing the CS gate both for the NISQ as well as fault-tolerant era. So one aim is to have some representation or formalism to express the unitaries implementable by this gate set and also to characterize them. We also intend to use these theoretical results in order to develop CS-optimal synthesis algorithms. These will help us compare the different universal gate sets, specifically the set of unitaries exactly implementable by these gate sets. Such questions are very relevant for resource estimations of various algorithms. Due to various reasons like the fidelity of gates, ease of implementation, etc, different hardware platforms may prefer different universal gate sets. The ability to compare the relative number of different non-Clifford gates required to implement a certain unitary with different universal gate sets, will help in making wiser decisions about which gate set to use^27,28^. Also, in literature some popular unitaries have implementations only in a specific gate set. We can then have a more convenient estimate on the non-Clifford gate count simply by replacing each such gate with the new gate set.

We develop both provable and heuristic algorithms. For the former type of algorithm there exists rigorous proof about the claimed optimality and complexity. For the latter the claims depend on some unproven conjectures. Usually when the complexity of the existing provable algorithms is very high such that they are nearly impractical to implement, then we take recourse to heuristics.

**Table 1:** Summary of space and time complexity of the different algorithms developed in this paper

Algorithm	Type of algorithm	Type of unitary	Time complexity	Space complexity
APPROX-CS-OPT	Provable	Approximately implementable	$O\left( n^{2m_{\epsilon }} 2^{(4n-8)m_{\epsilon }} \right)$	$O\left( n^22^{4n-8}\right)$
Nested MITM	Provable	Exactly implementable	$O\left( n^{2(c-1)\lceil \frac{m}{c}\rceil } 2^{(4n-8)(c-1)\lceil \frac{m}{c}\rceil } \right)$	$O\left( n^{2\lceil \frac{m}{c}\rceil }2^{(4n-8)\lceil \frac{m}{c}\rceil } \right)$
EXACT-CS-OPT	Heuristic	Exactly implementable	${\hbox {Poly}}(n^22^{4n-8},m)$	${\hbox {Poly}}(n^22^{4n-8},m)$

Here *m* and $m_{\epsilon }$ are the CS-count and $\epsilon$-CS-count of the input *n*-qubit unitary ($n > 0$), respectively. $c\ge 2$ is the level of nesting

1. We define a generating set ${\mathcal {G}}_{CS}$ such that any unitary exactly implementable by the Clifford+CS gate set can be written as product of unitaries from this gate set and a Clifford (Theorem 3.2 in section "Generating set"). We show that $|{\mathcal {G}}_{CS}|\in O\left( n^22^{4n-8} \right)$ (Theorem 3.3 in section "Generating set").
2. With the help of ${\mathcal {G}}_{CS}$ we derive a lower bound of $\Omega \left( \log _{\frac{4}{\sqrt{2}}}\left( \frac{1}{\epsilon }\right) \right)$ on the $\epsilon$-CS count of arbitrary multi-qubit unitaries (Theorems 3.5 and 3.6 in section "Bound on CS-count of unitaries"). For a unitary *W*, its $\epsilon$-CS count is the minimum number of CS gates required to implement any unitary that is within $\epsilon$ distance of *W*. Most of the previous works have proven bounds on the total gate count or 1-qubit non-Clifford gate count like T^10,13^ and V^9,11,12,29^. Most of these are upper bounds and work for 1-qubit gates, specifically z-rotation gates. The bound on T-count derived in^13^ is empirical, meaning it is interpolated from experimental data and there are no rigorous analysis. In fact, it is not clear how to extend the number-theoretic arguments used in^9–12^ for multi-qubit gates. For the special case of 2-qubit unitaries, an upper bound of $5\log _2\frac{1}{\epsilon }+O(1)$ on the CS-count, has been derived in^26^. A bound on the Toffoli-count of arbitrary multi-qubit unitaries has been derived in^30^.
3. We then derive the channel representation of unitaries in this set (Theorem 4.2 in section "Channel representation"), which helps us infer that the T gate cannot be exactly implemented by Clifford+CS (Theorem 4.5), while we know that CS can be exactly implemented with 3 T gates. This implies that ${\mathcal {J}}_n^{CS}\subset {\mathcal {J}}_n^{T}$, where ${\mathcal {J}}_n^{CS}$ and ${\mathcal {J}}_n^T$ are the set of unitaries exactly implementable by the Clifford+CS and Clifford+T gate set, respectively. One advantage of our approach in using channel representation in order to derive such exact implementability results is Fact 4.3, which ensures that a unitary is the channel representation of a Clifford operator if and only if it has exactly one $\pm 1$ in each row and column. This implies that to check implementability of a group of the form Clifford+non-Clifford we need to focus on the channel representation of the non-Clifford operators only. For example, we can show that CH cannot be exactly implemented by Clifford+Toffoli or Clifford+CS because its channel representation has $\pm \frac{1}{\sqrt{2}}$ which is outside the ring in which the entries of the channel representation of unitaries exactly implementable by Clifford+Toffoli or Clifford+CS belong (Lemma 4.4).
4. The channel representation also inherits the advantages already established in^32,33^, for designing algorithms. First, channel representation of ${\mathcal {G}}_{CS}$ helps us develop CS-count-optimal synthesis algorithms, using the nested meet-in-the-middle (MITM) algorithm, introduced in^32^. This algorithm was developed as a recursive generalization of the MITM algorithm of^31^. The space and time complexity of our algorithm (section "Implementation results") is $O\left( n^{2\lceil \frac{m}{c}\rceil }2^{(4n-8)\lceil \frac{m}{c}\rceil } \right)$ and $O\left( n^{2(c-1)\lceil \frac{m}{c}\rceil }2^{(4n-8)(c-1)\lceil \frac{m}{c}\rceil } \right)$, respectively. Here $m={\mathcal {S}}(U)$ is the CS-count of the input unitary *U* and $c\ge 2$ is the level of nesting used. Second, we define the smallest denominator exponent ($\text {sde}_2$) with respect to 2 and discuss how it can be used to design much more efficient heuristic algorithms (section "Implementation results"). The time and space complexity of our algorithm is $\text {poly}(n^22^{4n-8},{\mathcal {S}}(U))$. Third, we can develop clever data structures, such that no floating point operations are required at any step of our algorithms for exactly implementable unitaries. Fourth, we design a fast multiplication algorithm to multiply two channel representations, which basically boils down to some row additions involving part of the matrices. This is especially useful when we have to perform many such multiplications. With the help of these algorithms we prove that the optimal CS-count for Toffoli is 3. In^30^ we show that the minimum number of Toffoli gates required to implement CS is also 3. This implies that ${\mathcal {J}}_n^{Tof}={\mathcal {J}}_n^{CS}$, where ${\mathcal {J}}_n^{Tof}$ is the set of unitaries exactly implementable by the Clifford+Toffoli gate set. Previously, CS-count-optimal-synthesis algorithm for 2-qubit unitaries has been developed in^26^. Here we emphasize that we do not claim that we derive the first circuit for Toffoli with Clifford+CS. But with our algorithms we derive a CS-count-optimal one.
5. We design a provably CS-count-optimal synthesis algorithm for approximately implementable unitaries (section "CS-count-optimal synthesis algorithm for approximately implementable unitaries"), analogous to the one in^15^ for Clifford+T. The time complexity of this algorithm is $O\left( n^{2{\mathcal {S}}_{\epsilon }(W)}2^{(4n-8){\mathcal {S}}_{\epsilon }(W)} \right)$, where ${\mathcal {S}}_{\epsilon }(W)$ is the $\epsilon$-CS-count of the input unitary *W*. The space complexity is $O\left( n^22^{4n-8}\right)$. The $\epsilon$-CS-count of *W* is the minimum number of CS gates required to implement any unitary within distance $\epsilon$ of *W*. We have summarized the complexities of the various algorithms developed in this paper in Table 1.

### Relevant work

Much work has been done to characterize the unitaries exactly implementable by the various universal gate sets^34^ like Clifford+T^14,31,35^, V-basis^29^, Clifford+CS^26^ (2 qubits only), Toffoli+H^36^ and the more general Clifford+Toffoli^30^. Extensive work has been done to synthesize unitaries without optimality constraint on any particular gate^7,8,36–40^. More work has been done for the synthesis of V-count-optimal circuits^9,11,12^, but these work only for 1-qubit unitaries and the optimality is guaranteed only for the z-rotations. Recently in^41^ algorithms have been developed that synthesize V-count-optimal circuits for multi-qubit unitaries. Extensive work has been done for T-count-optimal synthesis of exactly implementable multi-qubit unitaries^31,32,42^ and approximately implementable 1-qubit unitaries^10,13,43^. The algorithms in^15,30^ are the only ones that synthesize T-count-optimal and Toffoli-count-optimal circuits for arbitrary multi-qubit unitaries, respectively.

The complexity of the optimal-synthesis algorithms discussed so far, cannot avoid the exponential dependence on *n* since the input being an *n*-qubit unitary has size $2^n\times 2^n$. If additional optimality constraints are imposed then complexity increases even further, and often it also exponentially depends on other parameters, for example the minimum non-Clifford gate count. Heuristics have been designed that reduces the dependence on these other parameters to polynomial^32^. However, with exponential dependence on *n*, these algorithms become intractable on a personal computer. So significant amount of work has been done to develop re-synthesis algorithms where the input is usually an already synthesized circuit and the output is a circuit with reduced gate count. Due to relaxed constraint and more input information, the complexity of these algorithms are polynomial in input size. However, in literature this reported complexity does not include the cost of generating the input circuit which itself is exponential in *n*. Usually, optimal-synthesis algorithms are used to synthesize smaller unitaries that are more frequent in quantum algorithms, for example rotations. One can do piece-wise optimal synthesis of a larger circuit and then estimate the non-Clifford gate cost.

## Preliminaries

*Notations :* We denote the $n\times n$ identity matrix by ${\mathbb {I}}_n$ or ${\mathbb {I}}$ if dimension is clear from the context. We denote the $(i,j)^{th}$ entry of a matrix *M* by *M*[*i*, *j*]. Here we note that we denote the commutator bracket by $[A,B]=AB-BA$, where *A*, *B* are operators. Though we use the same kind of brackets, the meaning should be clear from the context. We denote the $i^{th}$ row of *M* by *M*[*i*, .]. We denote the set of *n*-qubit unitaries by ${\mathcal {U}}_n$. The size of an *n*-qubit unitary is $N\times N$ where $N=2^n$. The qubit(s) on which a gate acts is (are) mentioned in the subscript with brackets. For example, $\text {X}_{(q)}$ implies an X gate acting on qubit *q*. For multi-qubit controlled gate the control qubit(s) is (are) mentioned first, followed by the target qubit(s), separated by semi-colon. For example, $\text {CNOT}_{i;j}$ denotes CNOT gate controlled on qubit *i* and target on qubit *j* and $\text {TOF}_{i,j;k}$ denotes the Toffoli gate with controls on qubits *i*, *j* and target on qubit *k*. For symmetric multi-qubit gates like CS, where the unitary does not change if we interchange the control and target qubit, we replace the semi-colon with a comma. For convenience we skip the subscript, when it is clear from the context.

We have given detail description about the n-qubit Pauli operators (${\mathcal {P}}_n$) and the Clifford group (${\mathcal {C}}_n$) in Appendix A.1. The Clifford+CS group is generated by ${\mathcal {C}}_n$ and the CS gate (Eq. 1). A unitary *U* is exactly implementable by the Clifford+CS gate set if there exists an implementing circuit (up to some global phase) consisting of these gates, else it is approximately implementable by this gate set. We use ${\mathcal {J}}_n^{CS}$ to denote the set of *n*-qubit unitaries exactly implementable by Clifford+CS. Specifically, we say *W* is $\epsilon$-approximately implementable by the Clifford+CS gate set if there exists an exactly implementable unitary *U* such that $d(U,W)\le \epsilon$. The Solovay-Kitaev algorithm^7,8^ guarantees that any unitary is $\epsilon$-approximately implementable, for arbitrary precision $\epsilon \ge 0$. In this paper we use the following distance measure *d*(., .), which has been used in previous works like^13,15,38^ (qubit based computing),^16,17^ (topological quantum computing).

### Definition 2.1

(*Global phase invariant distance*) Given two unitaries $U,W\in {\mathcal {U}}_n$, we define the global phase invariant distance between them as follows.

$$\begin{aligned} d(U,W)=\sqrt{1-\frac{\left| \text {Tr}\left( U^{\dagger }W\right) \right| }{N}} \end{aligned}$$

Like the operator norm, this distance is composable and inequalities relating these two distance metrics have been derived in^14^.

### CS-count of circuits and unitaries

#### CS-count of circuits

The *CS-count of a circuit* is the number of CS-gates in it.

#### CS-count of exactly implementable unitaries

The *CS-count of an exactly implementable unitary* *U*, denoted by ${\mathcal {S}}(U)$, is the minimum number of CS-gates required to implement it (up to a global phase) with a Clifford+CS circuit.

#### $\epsilon$-CS-count of approximately implementable unitaries

Let $W\in {\mathcal {U}}_n$ be an approximately implementable unitary. The $\epsilon$-*CS-count* of *W*, denoted by ${\mathcal {S}}_{\epsilon }(W)$, is the minimum number of CS gates required to implement any unitary that is within distance $\epsilon$ of *W*. In other words ${\mathcal {S}}_{\epsilon }(W) ={\mathcal {S}}(U)$, where $U\in {\mathcal {J}}_n^{CS}$ and ${\mathcal {S}}(U)\le {\mathcal {S}}(U')$ for any exactly implementable unitary $U'$ within distance $\epsilon$ of *W*. We call a CS-count-optimal circuit for *U* as the $\epsilon$-*CS-count-optimal* circuit for *W*. It is not hard to see that these definitions are very general and can be applied to any unitary $W\in {\mathcal {U}}_n$, exactly or approximately implementable. If a unitary is exactly implementable then $\epsilon =0$.

## Generating set

Any unitary *U* exactly implementable by Clifford+CS can be written as alternative product of a Clifford and a CS gate. We can alternatively express *U* as product of unitaries that are Clifford conjugation of CS gates.

2 $$\begin{aligned} U= & {} e^{i\phi }C_m({\widetilde{CS}})C_{m-1}({\widetilde{CS}})\ldots C_1({\widetilde{CS}})C_0;\qquad \left[ C_i\in {\mathcal {C}}_n,{\widetilde{CS}}\in \{CS,CS^{\dagger }\},\phi \in [0,2\pi ) \right] \nonumber \\= & {} e^{i\phi }\left( C_m({\widetilde{CS}})C_m^{\dagger }\right) \left( C_mC_{m-1}({\widetilde{CS}})C_{m-1}^{\dagger }C_{m}^{\dagger }\right) \ldots \left( C_m\ldots C_1({\widetilde{CS}})C_1^{\dagger }\ldots C_m^{\dagger }\right) C_m\ldots C_1C_0 \nonumber \\= & {} e^{i\phi }\left( \prod _{j=m}^1C_j'({\widetilde{CS}})C_j'^{\dagger }\right) C_0'\qquad [C_j'\in {\mathcal {C}}_n; j=0,\ldots ,m] \end{aligned}$$

Let $CS_{(i,j)}$ be a CS gate acting on qubits *i* and *j*. Then,

3 $$\begin{aligned} CS_{(i,j)}= & {} \left( \frac{3+i}{4}\right) ({\mathbb {I}}_{(i)}\otimes {\mathbb {I}}_{(j)})+\left( \frac{1-i}{4}\right) (\text {Z}_{(i)} +\text {Z}_{(j)} -\text {Z}_{(i)}\otimes \text {Z}_{(j)} ); \end{aligned}$$

and if $C\in {\mathcal {C}}_n$ is an *n*-qubit Clifford then,

4 $$\begin{aligned} C(CS_{(i,j)})C^{\dagger }= & {} \left( \frac{3+i}{4}\right) {\mathbb {I}}_n+\left( \frac{1-i}{4}\right) \left( C\text {Z}_{(i)}C^{\dagger }+C\text {Z}_{(j)}C^{\dagger }-(C\text {Z}_{(i)}C^{\dagger }) (C\text {Z}_{(j)}C^{\dagger }) \right) \nonumber \\= & {} \beta _0{\mathbb {I}}_n+\beta _1(P_1+P_2-P_1P_2):= G_{P_1,P_2}; \end{aligned}$$

where $\beta _0=\frac{3+i}{4}$, $\beta _1=\frac{1-i}{4}$, $P_1=C\text {Z}_{(i)}C^{\dagger }$, $P_2=C\text {Z}_{(j)}C^{\dagger }$. Since $[Z_{(i)},\text {Z}_{(j)}]=0$, so using Fact A.4 we have $[P_1,P_2]=0$. Also $P_1\ne P_2$ and $P_1,P_2\ne {\mathbb {I}}$. In Appendix B (Lemma B.1) we have given detailed proof about the following properties of the unitaries $G_{P_1,P_2}$.

### Lemma 3.1

If $P_1,P_2\in {\mathcal {P}}_n\setminus \{{\mathbb {I}}_n\}$ such that $[P_1,P_2]=0$, then

1. $$G_{P_1,P_2}=G_{P_2,P_1}$$;
2. $$G_{P_1,-P_1P_2}=G_{P_1,P_2}$$;
3. $G_{P_1,-P_2}=G_{P_1,P_2}C$, where $C\in {\mathcal {C}}_n$ is an *n*-qubit Clifford.

From Eq. (2) and using the above lemma we can define the following set, which we call a generating set (modulo Clifford), because we can express any exactly implementable unitary (up to a global phase) as product of unitaries from this set and a Clifford.

5 $$\begin{aligned} {\mathcal {G}}_{CS}=\left\{ G_{P_1P_2}: P_1,P_2\in {\mathcal {P}}_n\setminus \{{\mathbb {I}}\};\quad P_1\ne P_2;\quad [P_1,P_2]=0;\quad (P_1,P_2)\equiv (P_2,P_1)\equiv (P_1,\pm P_1P_2) \right\} \end{aligned}$$

We use $(P_1,P_2)\equiv (P_1',P_2')$ to imply that only one pair is included in the set. In Algorithm 1 (Appendix E) we have given the pseudocode for constructing this set. Specifically, we have the following result.

### Theorem 3.2

Any unitary *U* that is exactly implementable by the Clifford+CS gate set can be expressed as,

$$\begin{aligned} U=e^{i\phi } \left( \prod _{j=m}^1 G_{P_{1j},P_{2j} } \right) C_0;\qquad [C_0\in {\mathcal {C}}_n; \phi \in [0,2\pi ) ]; \end{aligned}$$

where $G_{P_{1j},P_{2j}} \in {\mathcal {G}}_{CS}$, a set defined in Eq. (5).

*Cardinality of* ${\mathcal {G}}_{CS}$: We prove the following bound on $|{\mathcal {G}}_{CS}|$, using some combinatorial arguments on the set of Paulis. The proof has been given in Appendix B (Theorem B.2).

### Theorem 3.3

$$\begin{aligned} |{\mathcal {G}}_{CS}|\le \frac{1}{8}(16^n-13^n-4^n+1)+\frac{1}{12}(12^n-2\cdot 6^n) \in O\left( n^216^{n-2} \right) \end{aligned}$$

For $n=2$, we obtain $|{\mathcal {G}}_{CS}|=15$, which is also equal to the set derived in^26^. In this paper the authors derived a generating set for the special case of 2-qubit unitaries. We emphasize that our methods are completely different from this paper and is more general and works for any $n > 0$. In Table 2 we have given the cardinality of the generating set obtained in practice, for different number of qubits, using Algorithm 1 (Appendix E).

**Table 2:** Cardinality of ${\mathcal {G}}_{CS}$ for different number of qubits *n*

*n*	2	3	4	5
$|{\mathcal {G}}_{CS}|$	15	315	5355	86955
Time	0.006 s	1.787 s	2 min 16 s	6 h 11 min

*Circuit construction for* $G_{P_1,P_2}$: We can construct a circuit implementing $G_{P_1,P_2}$ by deriving the conjugating Clifford and determining the control and target qubits of the CS gate. Given a pair of commuting non-identity Paulis $P_1,P_2$, we use the algorithm in^44^ in order to derive a conjugating Clifford $C'\in {\mathcal {C}}_n$ such that $C'P_1C'^{\dagger }=P_1'$ and $C'P_2C'^{\dagger }=P_2'$, where $P_1'=\bigotimes _{j=1}^nQ_j$, $P_2'=\bigotimes _{j=1}^nR_j$ and $Q_j,R_j\in \{{\mathbb {I}},\text {Z}\}$. That is, the output of this algorithm is a pair of $\text {Z}$-operators i.e. *n*-qubit Paulis that are tensor product of either ${\mathbb {I}}$ or $\text {Z}$. Then we use the following conjugation relations,

$$\begin{aligned}{} & {} \text {SWAP}({\mathbb {I}}\otimes \text {Z})\text {SWAP}=\text {Z}\otimes {\mathbb {I}};\quad \text {CNOT}_{(j;k)} ({\mathbb {I}}_{(j)}\otimes \text {Z}_{(k)})\text {CNOT}_{(j;k)}=\text {Z}_{(j)}\otimes \text {Z}_{(k)}\\{} & {} \text {CNOT}_{(j;k)} (\text {Z}_{(j)}\otimes {\mathbb {I}}_{(k)})\text {CNOT}_{(j;k)}=\text {Z}_{(j)}\otimes {\mathbb {I}}_{(k)}; \end{aligned}$$

in order to derive Clifford $C''\in {\mathcal {C}}_n$ such that $C''P_1'C''^{\dagger }=\text {Z}_{(a)}$ and $C''P_2'C''^{\dagger }=\text {Z}_{(b)}$, where $1\le a,b\le n$ and $a\ne b$. If $C=C'C''$, then we get $C'C''\text {Z}_{(a)}C''^{\dagger }C'^{\dagger }=P_1$ and $C'C''\text {Z}_{(b)}C''^{\dagger }C'^{\dagger }=P_2$. Thus a circuit for $G_{P_1,P_2}$ consists of $\text {CS}_{(a,b)}$, conjugated by Clifford $C=C'C''$.

### Bound on CS-count of unitaries

In this section we derive lower bound on the CS-count of arbitrary *n*-qubit unitaries. We know that any unitary can be expanded in the Pauli basis. Let *W* be an *n*-qubit unitary and

6 $$\begin{aligned} W = \sum _{i=1}^{4^n} \overline{\alpha _i}P_i \qquad [P_i\in {\mathcal {P}}_n]. \end{aligned}$$

*Not exactly implementable:* First we consider the case when *W* is not exactly implementable. Let $U = \left( \prod _{j=1}^mG_{P_{1_j},P_{2_j}} \right) C_0e^{i\phi }$ be a unitary such that $W = UE$, for some $C_0\in {\mathcal {C}}_n$, $\phi \in [0,2\pi )$ and unitary *E*. Also $d(U, W) \le \epsilon$ implying $|\text {Tr}(E)|\ge N(1-\epsilon ^2)$, where $N=2^n$ and $m={\mathcal {S}}_{\epsilon }(W)$. Let ${\widetilde{U}} = \prod _{j=1}^mG_{P_{1_j},P_{2_j}}$. Then,

7 $$\begin{aligned} \left| \text {Tr}\left( {\widetilde{U}} W^{\dagger } \right) \right| = \left| \text {Tr}(EC_0) \right| \end{aligned}$$

and if $C_0=\sum _{P\in {\mathcal {P}}_n} r_PP$, $M=\left| \left\{ P:r_P \ne 0 \right\} \right|$, then from Theorem 3.1 of^15^ we have the following.

8 $$\begin{aligned}{} & {} \frac{1-\epsilon ^2}{\sqrt{M}} - \sqrt{M(2\epsilon ^2-\epsilon ^4)} \le | \text {Tr}(EC_0P'/N) | \le \frac{1}{\sqrt{M}} + \sqrt{M(2\epsilon ^2-\epsilon ^4)} \quad [\text {if }\quad r_{P'}\ne 0] \end{aligned}$$

9 $$\begin{aligned}{} & {} 0 \le | \text {Tr}(EC_0P'/N) | \le \sqrt{M(2\epsilon ^2-\epsilon ^4)} \quad [\text {if }\quad r_{P'} = 0] \end{aligned}$$

here $1\le M\le N^2$. We use this in order to lower bound $m={\mathcal {S}}_{\epsilon }(W)$ as a function of $\epsilon$. First we observe that we can expand ${\widetilde{U}}$ as in the following lemma, the proof of which has been given in Appendix C.

#### Lemma 3.4

If ${\widetilde{U}}= \prod _{j=1}^mG_{P_{1_j},P_{2_j}}$, where $G_{P_{1_j},P_{2_j}} = a{\mathbb {I}}+bQ_j$, $Q_j = P_{1_j}+P_{2_j}-P_{1_j}P_{2_j}$, $a = \frac{3+i}{4}$ and $b = \frac{1-i}{4}$, then

$$\begin{aligned} {\widetilde{U}}= & {} a^m{\mathbb {I}}+a^{m-1}b\left( \sum _{j}Q_j\right) +a^{m-2}b^2\left( \sum _{j_1< j_2} Q_{j_1}Q_{j_2} \right) +a^{m-3}b^3\left( \sum _{j_1< j_2< j_3}Q_{j_1}Q_{j_2}Q_{j_3} \right) +\cdots \\{} & {} \cdots +ab^{m-1} \left( \sum _{j_1< j_2< \cdots < j_{m-1}} Q_{j_1}Q_{j_2}\cdots Q_{j_{m-1}} \right) + b^m \prod _{j=1}^mQ_j \end{aligned}$$

Now from Eqs. (6) and (7) we have

$$\begin{aligned} | \text {Tr}(EC_0) | = \left| \text {Tr}\left( {\widetilde{U}}W^{\dagger } \right) \right| = \left| \sum _{i=1}^{4^n}\alpha _i{\widetilde{U}}P_i \right| , \end{aligned}$$

and thus using triangle inequality and Eqs. (9) and (8), we have

10 $$\begin{aligned} \sum _{i=1}^{4^n} |\alpha _i| |\text {Tr}({\widetilde{U}}P_i P'/N)|\le & {} \frac{1}{\sqrt{M}} +\sqrt{M(2\epsilon ^2-\epsilon ^4)} \le \frac{1}{\sqrt{M}} +\sqrt{2M}\epsilon \end{aligned}$$

11 $$\begin{aligned} \left| |\alpha _k| |\text {Tr}({\widetilde{U}}P_kP'/N)| - \sum _{i\ne k}|\alpha _i| |\text {Tr}({\widetilde{U}}P_i P'/N)| \right|\ge & {} \frac{1-\epsilon ^2}{\sqrt{M}} -\sqrt{M(2\epsilon ^2-\epsilon ^4)} \ge \frac{1-\epsilon ^2}{\sqrt{M}} -\sqrt{2M}\epsilon \nonumber \\ -\left| |\alpha _k| |\text {Tr}({\widetilde{U}}P_kP'/N)| - \sum _{i\ne k}|\alpha _i| |\text {Tr}({\widetilde{U}}P_i P'/N)| \right|\le & {} \frac{-1+\epsilon ^2}{\sqrt{M}} +\sqrt{2M}\epsilon . \end{aligned}$$

Without loss of generality let us assume that $|\alpha _k| |\text {Tr}({\widetilde{U}}P_kP'/N)| - \sum _{i\ne k}|\alpha _i| |\text {Tr}({\widetilde{U}}P_i P'/N)| > 0$. So, adding the above inequalities we get

$$\begin{aligned} \sum _{i\ne k} |\alpha _i| |\text {Tr}({\widetilde{U}}P_i P' /N )| \le \frac{\epsilon ^2}{2\sqrt{M}} + \sqrt{2M}\epsilon . \end{aligned}$$

Now $\sum _{i\ne k} |\alpha _i| |\text {Tr}({\widetilde{U}}P_i P' /N )| \ge |\alpha _{\ell }| |\text {Tr}({\widetilde{U}}P_{\ell } P' /N )|$, for some $P_{\ell }$ such that the absolute value of coefficients on the right hand side is non-zero. For $M\ge 1$ and non-Clifford ${\widetilde{U}}$, $EC_0$ there exists one such $P_{\ell }$. So,

12 $$\begin{aligned} |\alpha _{\ell }| |\text {Tr}({\widetilde{U}}P_{\ell } P' /N )| \le \frac{\epsilon ^2}{2\sqrt{M}} + \sqrt{2M}\epsilon . \end{aligned}$$

Now we observe that $\text {Tr}({\widetilde{U}} P_{\ell }P'/N)$ is the coefficient of $P_{\ell }P'$ in the Pauli expansion of ${\widetilde{U}}$, as given in Lemma 3.4. Consider group of r-product terms of the form $a^{m-r}b^r \left( \sum _{j_1<j_2<\cdots <j_r} Q_{j_1}Q_{j_2}\ldots Q_{j_r} \right)$. We remember that each $Q_j$ is a linear combination of 3 Paulis and $Q_j^2=3{\mathbb {I}}-2Q_j$ (Equation 42 in Appendix D), which implies that $Q^m$ is a linear combination of 4 Paulis, for any $m\ge 2$. Thus each summand in a r-product group is a product of at most *r* Paulis. The expression of ${\widetilde{U}}$ is sum of $m+1$ such groups of *r*-products, where each combination is distinct from the other. Overall, there can be at least a constant number of terms that are $P_{\ell }P'$. Since $|b|<|a|<1$ so minimum value of the coefficient can be $c|b|^m$, where *c* is a constant. Thus from Eq. (12),

$$\begin{aligned} |\alpha _{\ell }| c|b|^m\le & {} |\alpha _{\ell }| |\text {Tr}({\widetilde{U}}P_{\ell } P' /N )| \le \frac{\epsilon ^2}{\sqrt{2M}} +\sqrt{2M}\epsilon = \epsilon \left( \frac{\epsilon }{2\sqrt{M}} +\sqrt{2M} \right) . \\ |b|^m\le & {} \frac{\epsilon }{c|\alpha _{\ell }|} \left( \frac{\epsilon }{2\sqrt{M}} +\sqrt{2M} \right) \\ m \log \frac{1}{|b|}\ge & {} \log \frac{1}{\epsilon } + \log \left( \frac{ c|\alpha _{\ell }| }{ \frac{\epsilon }{2\sqrt{M}} +\sqrt{2M} } \right) \\ m\ge & {} \log _{\frac{1}{|b|}} \left( \frac{1}{\epsilon } \right) - \log _{\frac{1}{|b|}} \left( \frac{ \frac{\epsilon }{2\sqrt{M}} +\sqrt{2M} }{ c|\alpha _{\ell }| } \right) \end{aligned}$$

Since $|b|=\frac{\sqrt{2}}{4}$, we have the following asymptotic lower bound with respect to $\epsilon$.

#### Theorem 3.5

Let $W = \sum _i\alpha _iP_i$ be an *n*-qubit unitary approximately implementable by the Clifford+CS gate set. Here $P_i\in {\mathcal {P}}_n$. Then for some constant *c*,

$$\begin{aligned} {\mathcal {S}}_{\epsilon }\left( W\right)\ge & {} \log _{\frac{4}{\sqrt{2}}} \left( \frac{1}{\epsilon } \right) - \log _{\frac{4}{\sqrt{2}}} \left( \frac{ \frac{\epsilon }{2\sqrt{M}} +\sqrt{2M} }{ c|\alpha _{max}| } \right) \in \Omega \left( \log _{\frac{4}{\sqrt{2}}} \left( \frac{1}{\epsilon } \right) \right) . \end{aligned}$$

*Exactly implementable : * Now we consider the case when *W* is exactly implementable i.e. $E={\mathbb {I}}$ and $\epsilon = 0$. Then from Fact A.3 in Appendix A^45^ or plugging in $\epsilon = 0$ in Eq. (9), we obtain

$$\begin{aligned} \sum _{i=1}^{4^n} |\alpha _i| |\text {Tr}({\widetilde{U}}P_i P'/N)|\le & {} \frac{1}{\sqrt{M}}. \end{aligned}$$

Now, $|\alpha _{\ell }| |\text {Tr}({\widetilde{U}}P_{\ell } P'/N)| \le \sum _{i=1}^{4^n} |\alpha _i| |\text {Tr}({\widetilde{U}}P_i P'/N)|$ , where $|\alpha _{\ell }|\ne 0$. As previously argued $c|b|^m\le |\text {Tr}({\widetilde{U}}P_{\ell } P'/N)|$, for some constant *c*. So,

13 $$\begin{aligned} |\alpha _{\ell }| c|b|^m\le & {} \frac{1}{\sqrt{M}} \nonumber \\ \left( \frac{1}{|b|}\right) ^m\ge & {} |\alpha _{\ell }|c\sqrt{M} \nonumber \\ \implies m\ge & {} \log _{\frac{1}{|b|}} \left( |\alpha _{max}|c\sqrt{M} \right) . \end{aligned}$$

Thus we have the following result.

#### Theorem 3.6

Let $W = \sum _i\alpha _iP_i$ be an *n*-qubit unitary exactly implementable by the Clifford+CS gate set. Here $P_i\in {\mathcal {P}}_n$. Then for some constant *c* and $1\le M\le 4^n$,

$$\begin{aligned} {\mathcal {S}}\left( W\right)\ge & {} \log _{\frac{4}{\sqrt{2}}} \left( |\alpha _{max}|c\sqrt{M} \right) . \end{aligned}$$

*Upper bound : * An upper bound on the CS-count can be derived from the Solovay-Kitaev theorem^7,8,37^, which gives an upper bound of $O\left( \log ^c\left( \frac{1}{\epsilon }\right) \right)$ on the total number of gates, where $3\le c\le 4$ is a constant. Till now the best bound on *c* is $1.44042+\delta$^46^. This trivially implies an upper bound on the non-Clifford gate count, as well. Our lower bound in Theorem 3.5 is sub-quadratically smaller than this upper bound. For the special case of 2-qubit unitaries, an upper bound of $5\log _2\frac{1}{\epsilon }+O(1)$ has been derived in^26^. Our lower bound in this special case is within constant factor of this bound.

#### Illustration: CS-count of rotation gates

We consider the following rotation gates. In Appendix C.1 we have explicitly derived the Pauli basis expansions for each unitary. These are some standard unitaries that have been widely studied in previous literature.

14 $$\begin{aligned} R_z(\theta )= & {} e^{-i\frac{\theta }{2}} \begin{bmatrix} 1 &{} 0 \\ 0 &{} e^{i\theta } \end{bmatrix} = e^{-i\frac{\theta }{2}} \left( \alpha _1{\mathbb {I}}+\alpha _2\text {Z}\right) \nonumber \\ {\mathbb {I}}\otimes R_z(\theta )= & {} e^{-i\frac{\theta }{2}} \left( \alpha _1 {\mathbb {I}}{\mathbb {I}}+\alpha _2{\mathbb {I}}\text {Z}\right) \end{aligned}$$

here $\alpha _1 = \frac{1+e^{i\theta }}{2}$, $\alpha _2 = \frac{1-e^{i\theta }}{2}$ and so $|\alpha _1| = \cos \frac{\theta }{2}$, $|\alpha _2| = \sin \frac{\theta }{2}$.

Next, we consider a controlled $R_z(\theta )$ unitary, as defined below.

15 $$\begin{aligned} cR_z(\theta )= & {} \begin{bmatrix} 1 &{} 0 &{} 0 &{} 0 \\ 0 &{} 1 &{} 0 &{} 0 \\ 0 &{} 0 &{} e^{-i\theta /2} &{} 0 \\ 0 &{} 0 &{} 0 &{} e^{i\theta /2} \end{bmatrix} = \alpha _1'{\mathbb {I}}{\mathbb {I}}+\alpha _2'\text {Z}{\mathbb {I}}+\alpha _3'{\mathbb {I}}\text {Z}+\alpha _4'\text {Z}\text {Z} \end{aligned}$$

here $\alpha _1' = \frac{1+\cos \frac{\theta }{2}}{2}$, $\alpha _2'=\frac{1-\cos \frac{\theta }{2}}{2}$, $\alpha _3' = \frac{i}{2}\sin \frac{\theta }{2}$, $\alpha _4' = -\alpha _3'$. So, $|\alpha _1'|=\frac{1+\cos \frac{\theta }{2}}{2}$, $|\alpha _2'|=\frac{1-\cos \frac{\theta }{2}}{2}$ and $|\alpha _3'| = |\alpha _4'| = \frac{\sin \frac{\theta }{2}}{2}$ .

Next, we consider the following controlled rotation gate, that is widely used in Quantum Fourier Transform^2^.

16 $$\begin{aligned} cR_n(\theta )= & {} \begin{bmatrix} 1 &{} 0 &{} 0 &{} 0 \\ 0 &{} 1 &{} 0 &{} 0 \\ 0 &{} 0 &{} 1 &{} 0 \\ 0 &{} 0 &{} 0 &{} e^{i\theta } \end{bmatrix} = \alpha _1''{\mathbb {I}}{\mathbb {I}}+\alpha _2''{\mathbb {I}}\text {Z}+\alpha _3''\text {Z}{\mathbb {I}}+\alpha _4''\text {Z}\text {Z} \end{aligned}$$

here $\alpha _1'' = \frac{3+e^{i\theta }}{4}$, $\alpha _2''=\alpha _3''=\frac{1-e^{i\theta }}{4}$, $\alpha _4'' = -\alpha _2''$. So, $|\alpha _1''|=\frac{1}{2}\sqrt{1+3\cos ^2\frac{\theta }{2}}$ and $|\alpha _2''| = |\alpha _3''| = |\alpha _4''|=\frac{1}{2}\sin \frac{\theta }{2}$.

Next, we consider the 2-qubit Given’s rotation unitary, that is used in quantum chemistry^47,48^, quantum simulations^49,50^, quantum machine learning^51^, variational quantum algorithms^52^.

$$\begin{aligned} Givens(\theta )= & {} \begin{bmatrix} 1 &{} 0 &{} 0 &{} 0 \\ 0 &{} \cos (\theta ) &{} -\sin (\theta ) &{} 0 \\ 0 &{} \sin (\theta ) &{} \cos (\theta ) &{} 0 \\ 0 &{} 0 &{} 0 &{} 1 \end{bmatrix} = \alpha _1'''{\mathbb {I}}{\mathbb {I}}+\alpha _2'''\text {X}\text {Y}+\alpha _3'''\text {Y}\text {X}+\alpha _4'''\text {Z}\text {Z}\end{aligned}$$

here $\alpha _1''' = \frac{1+\cos \theta }{2}$, $\alpha _2'''=i\frac{\sin \theta }{2}$, $\alpha _3'''=-i\frac{\sin \theta }{2}$ $\alpha _4''' = \frac{1-\cos \theta }{2}$. So, $|\alpha _1'''|=\frac{1+\cos \theta }{2}$, $|\alpha _2'''| = |\alpha _3'''| = \frac{\sin \theta }{2}$ and $|\alpha _4'''| = \frac{1-\cos \theta }{2}$.

We use $\text {diag}(d_1,d_2,\ldots ,d_N)$ to denote a $N\times N$ diagonal matrix with $d_1, d_2,\ldots ,d_N$ as diagonal entries, and 0 in the remaining places. Now we consider the following rotation gates, controlled on 2 qubits.

17 $$\begin{aligned} ccR_n(\theta )= & {} \text {diag}\left( 1,1,1,1,1,1,1,e^{i\theta } \right) \nonumber \\= & {} \widehat{\alpha _1}{\mathbb {I}}{\mathbb {I}}{\mathbb {I}}+ \widehat{\alpha _2}\text {Z}{\mathbb {I}}{\mathbb {I}}+\widehat{\alpha _3}{\mathbb {I}}\text {Z}{\mathbb {I}}+\widehat{\alpha _4}{\mathbb {I}}{\mathbb {I}}\text {Z}+\widehat{\alpha _5}\text {Z}\text {Z}{\mathbb {I}}+\widehat{\alpha _6}{\mathbb {I}}\text {Z}\text {Z}+\widehat{\alpha _7}\text {Z}{\mathbb {I}}\text {Z}+\widehat{\alpha _8}\text {Z}\text {Z}\text {Z} \end{aligned}$$

here $\widehat{\alpha _1}=\left( \frac{7+e^{i\theta }}{8}\right)$, $\widehat{\alpha _2}=\widehat{\alpha _3}=\widehat{\alpha _4}=\widehat{\alpha _8}=\left( \frac{1-e^{i\theta }}{8}\right)$, $\widehat{\alpha _5}=\widehat{\alpha _6}=\widehat{\alpha _7}=-\widehat{\alpha _8}$ and so, $|\widehat{\alpha _1}| =\frac{1}{4}\sqrt{9+7\cos ^2\frac{\theta }{2}}$, $|\widehat{\alpha _j}| =\frac{1}{4}\sin \frac{\theta }{2}$ when $j\ne 1$.

Finally, we consider the following rotation gate.

18 $$\begin{aligned} ccR_z(\theta )= & {} \text {diag}\left( 1,1,1,1,1,1,e^{-i\theta /2},e^{i\theta /2} \right) \nonumber \\= & {} \widetilde{\alpha _1}{\mathbb {I}}{\mathbb {I}}{\mathbb {I}}+ \widetilde{\alpha _2}\text {Z}{\mathbb {I}}{\mathbb {I}}+\widetilde{\alpha _3}{\mathbb {I}}\text {Z}{\mathbb {I}}+\widetilde{\alpha _4}{\mathbb {I}}{\mathbb {I}}\text {Z}+\widetilde{\alpha _5}\text {Z}\text {Z}{\mathbb {I}}+\widetilde{\alpha _6}{\mathbb {I}}\text {Z}\text {Z}+\widetilde{\alpha _7}\text {Z}{\mathbb {I}}\text {Z}+\widetilde{\alpha _8}\text {Z}\text {Z}\text {Z} \end{aligned}$$

here $\widetilde{\alpha _1}=\frac{3+\cos \frac{\theta }{2} }{4}$, $\widetilde{\alpha _2} = \widetilde{\alpha _3} = \frac{1-\cos \frac{\theta }{2}}{4}$, $\widetilde{\alpha _5}=-\widetilde{\alpha _2}$, $\widetilde{\alpha _6} = \widetilde{\alpha _7}= i\frac{\sin \frac{\theta }{2}}{4}$ and $\widetilde{\alpha _4}=\widetilde{\alpha _8}=-\widetilde{\alpha _6}$, and so,

$$\begin{aligned} |\widetilde{\alpha _j}|= & {} \frac{3+\cos \frac{\theta }{2} }{4}\qquad [j=1] \\= & {} \frac{1-\cos \frac{\theta }{2}}{4}\qquad [j=2,3,5] \\= & {} \frac{\sin \frac{\theta }{2}}{4}\qquad [j = 4,6,7,8]. \end{aligned}$$

Since each of these are approximately implementable unitaries, so we can obtain a lower bound on their $\epsilon$-CS-count by plugging in the respective values in Theorem 3.5.

## Channel representation

An *n*-qubit unitary *U* can be completely determined by considering its action on a Pauli $P_s\in {\mathcal {P}}_n : P_s\rightarrow UP_sU^{\dagger }$. The set of all such operators (with $P_s\in {\mathcal {P}}_n$) completely determines *U* up to a global phase. Since ${\mathcal {P}}_n$ is a basis for the space of all Hermitian $2^n\times 2^n$ matrices we can write

19 $$\begin{aligned} UP_sU^{\dagger }= & {} \sum _{P_r\in {\mathcal {P}}_n}\widehat{U}_{rs}P_r,\qquad \text {where }\quad \widehat{U}_{rs}=\frac{1}{2^n}\text {Tr}\left( P_rUP_sU^{\dagger }\right) . \end{aligned}$$

This defines a $4^n\times 4^n$ matrix $\widehat{U}$ with rows and columns indexed by Paulis $P_r,P_s\in {\mathcal {P}}_n$. We refer to $\widehat{U}$ as the channel representation of *U*. By Hermitian conjugation each entry of $\widehat{U}$ is real. The channel representation respects matrix multiplication and tensor product i.e. $\widehat{UW}=\widehat{U}\widehat{W}$ and $\left( \widehat{U\otimes W}\right) =\widehat{U}\otimes \widehat{W}$. Setting $V=U^{\dagger }$ it follows that $\widehat{U^{\dagger }}=\left( \widehat{U}\right) ^{\dagger }$, and we see that the channel representation $\widehat{U}$ is unitary.

### Fact 4.1

The channel representation inherits the decomposition from Theorem 3.2 (and in this decomposition there is no global phase factor).

$$\begin{aligned} \widehat{U}=\left( \prod _{j=m}^1\widehat{G_{P_{1j},P_{2j}}}\right) \widehat{C_0}\qquad [G_{P_{1j},P_{2j}}\in {\mathcal {G}}_{CS}] \end{aligned}$$

A decomposition in which $m={\mathcal {S}}(U)$ is called a CS-count-optimal decomposition. In the following theorem we summarize some important properties of ${\mathcal {G}}_{CS} = \left\{ \widehat{G_{P_{1_j},P_{2_j}}} : G_{P_{1_j},P_{2_j}} \in {\mathcal {G}}_{CS} \right\}$. These help us draw some important conclusions about the inexact implementability of some important unitaries. Also, they facilitate the design of proper data structure and efficient algorithms for matrix operations like multiplication, inverse, involving only integer arithmetic. Also we get the intuition to develop clever heuristics for CS-count-optimal synthesis algorithms.

### Theorem 4.2

Let $G_{P_1,P_2}\in {\mathcal {G}}_{CS}$, where $P_1,P_2\in {\mathcal {P}}_n\setminus \{{\mathbb {I}}\}$. Then its channel representation $\widehat{G_{P_1,P_2}}$ has the following properties.

1. The diagonal entries are 1 or $\frac{1}{2}$.
2. If a diagonal entry is 1 then all other entries in the corresponding row and column is 0.
3. If a diagonal entry is $\frac{1}{2}$ then there exists three entries in the corresponding row and column that are equal to $\pm \frac{1}{2}$, rest is 0.
4. Exactly $2^{2n-2}$ i.e. $\frac{1}{4}^{th}$ of the diagonal elements are 1, while the remaining are $\frac{1}{2}$.

The proof has been given in Appendix D. Since Cliffords map Paulis to Paulis, up to a possible phase factor of -1, so we have the following.

### Fact 4.3

(^31^) Let $\widehat{{\mathcal {C}}_n}=\{\widehat{C}:C\in {\mathcal {C}}_n\}$. A unitary $Q\in \widehat{{\mathcal {C}}_n}$ if and only if it has one non-zero entry in each row and column, equal to $\pm 1$.

The above fact, along with Theorem 4.2 and Fact 4.1, implies the following.

### Lemma 4.4

If *U* is a unitary exactly implementable by the Clifford+CS gate set, then each entry of $\widehat{U}$ belongs to the ring ${\mathbb {Z}}\left[ \frac{1}{2}\right] =\{\frac{a}{2^k} : a\in 2{\mathbb {Z}}+1, k\in {\mathbb {N}}\}$.

Since the channel representation of the non-Clifford T gate does not belong to this ring^32^, so it cannot be exactly implemented by Clifford+CS. On the other hand, we know that CS can be implemented exactly by 3 T gates. Thus every unitary exactly implementable by the Clifford+CS gate set can be exactly implemented by the Clifford+T gate set. But there exists unitaries exactly implementable by the Clifford+T gate set that cannot be exactly implemented by the Clifford+CS gate set. So the following theorem follows.

### Theorem 4.5

Let ${\mathcal {J}}_n^T$ and ${\mathcal {J}}_n^{CS}$ be the set of unitaries exactly implementable by the Clifford+T and Clifford+CS gate sets, respectively. Then ${\mathcal {J}}_n^{CS} \subset {\mathcal {J}}_n^T.$

In a separate paper^41^ we show that the entries of the channel representation of unitaries exactly implementable by the Clifford+V gate set, belongs to the ring ${\mathbb {Z}}\left[ \frac{1}{\sqrt{5}}\right]$. Thus, the V-basis gates are not exactly implementable by Clifford+CS and in fact, CS cannot be implemented exactly by this basis set, either. In section "Implementation results" we show that the minimum number of CS gates required to implement Toffoli, defined below, is 3.

20 $$\begin{aligned} Toffoli=\begin{bmatrix} 1 &{} 0 &{} 0 &{} 0 &{} 0 &{} 0 &{} 0 &{} 0 \\ 0 &{} 1 &{} 0 &{} 0 &{} 0 &{} 0 &{} 0 &{} 0 \\ 0 &{} 0 &{} 1 &{} 0 &{} 0 &{} 0 &{} 0 &{} 0 \\ 0 &{} 0 &{} 0 &{} 1 &{} 0 &{} 0 &{} 0 &{} 0 \\ 0 &{} 0 &{} 0 &{} 0 &{} 1 &{} 0 &{} 0 &{} 0 \\ 0 &{} 0 &{} 0 &{} 0 &{} 0 &{} 1 &{} 0 &{} 0 \\ 0 &{} 0 &{} 0 &{} 0 &{} 0 &{} 0 &{} 0 &{} 1 \\ 0 &{} 0 &{} 0 &{} 0 &{} 0 &{} 0 &{} 1 &{} 0 \end{bmatrix}, \end{aligned}$$

In a very recent paper^30^ we show that the minimum number of Toffoli gates required to implement CS is 3. Thus we have the following theorem.

### Theorem 4.6

Let ${\mathcal {J}}_n^{CS}$ and ${\mathcal {J}}_n^{Tof}$ be the set of unitaries exactly implementable by the Clifford+CS and Clifford+Toffoli gate sets, respectively. Then ${\mathcal {J}}_n^{Tof} = {\mathcal {J}}_n^{CS}.$

Here we define the following.

### Definition 4.7

For any non-zero $v\in {\mathbb {Z}}\left[ \frac{1}{2}\right]$ the smallest 2-denominator exponent, denoted by $\text {sde}_2$, is the smallest $k\in {\mathbb {N}}$ for which $v=\frac{a}{2^k}$, where $a\in 2{\mathbb {Z}}+1$.

We define $\text {sde}_2(0)=0$. For a $d_1\times d_2$ matrix *M* with entries over this ring we define

$$\begin{aligned} \text {sde}_2(M)= & {} \max _{a\in [d_1],b\in [d_2]}\text {sde}_2(M_{ab}). \end{aligned}$$

### Fact 4.8

Let $v_1,v_2,v_3,v_4\in {\mathbb {Z}}\left[ \frac{1}{2}\right]$ such that $\max \{\text {sde}_2(v_1),\text {sde}_2(v_2),\text {sde}_2(v_3),\text {sde}_2(v_4)\}=k$. Then $\text {sde}_2\left( \frac{1}{2}(v_1\pm v_2\pm v_3\pm v_4)\right) =k+1$ or $\le k$.

### Proof

Let $v_1=\frac{a}{2^w}$, $v_2=\frac{b}{2^x}$, $v_3=\frac{c}{2^y}$ and $v_4=\frac{d}{2^z}$, where $a,b,c,d\in 2{\mathbb {Z}}+1$ and $w,x,y,z\in {\mathbb {N}}$. Without loss of generality let $\max \{ w,x,y,z \}=x$

$$\begin{aligned} \frac{1}{2}(v_1\pm v_2\pm v_3\pm v_4)= & {} \frac{1}{2^{x+1}}\left( a\cdot 2^{x-w}\pm b\pm c\cdot 2^{x-y}\pm d\cdot 2^{x-z}\right) \end{aligned}$$

If the numerator is odd then there is no further reduction of the fraction and $\text {sde}_2$ of the resultant sum is $k+1$. If the numerator is even then there is a reduction of the fraction and the resultant $\text {sde}_2$ is at most *k*. $\square$

We represent each element $v=\frac{a}{2^k}\in {\mathbb {Z}}\left[ \frac{1}{2}\right]$ by a pair [*a*, *k*] of integers. In this way, all arithmetic operations involving elements within this ring, for example, matrix multiplication can be done with integer arithmetic. We have given the pseudocode for some of these operations in Appendix E (Algorithms 3 and 4). This also implies that we do not require any floating point operation for our CS-count-optimal synthesis algorithms for exactly implementable unitaries.

*Compact representation of* $\widehat{G_{P_1,P_2}}$: It is sufficient to represent the $2^{2n}\times 2^{2n}$ matrix $\widehat{G_{P_1,P_2}}$ with an array of length $3\cdot 2^{2n-2}$. For brevity, we write $\widehat{G_{P_1,P_2}}[P_r,P_s]$ as $\widehat{G_{P_1,P_2}}[r,s]$. Each entry of this array is of the form $(r,\pm s, \pm s', \pm s'' )$, which implies $\widehat{G_{P_1,P_2}}[r,r]=\frac{1}{2}$, $\widehat{G_{P_1,P_2}}[r,s]=\pm \frac{1}{2}$, $\widehat{G_{P_1,P_2}}[r,s']=\pm \frac{1}{2}$ and $\widehat{G_{P_1,P_2}}[r,s'']=\pm \frac{1}{2}$. The remaining entries of the matrix can be easily deduced, using Theorem 4.2. In Algorithm 2 (Appendix E) we have provided a pseudocode for computing the channel representation of these basis elements and storing them using this compact representation.

*Channel representation of* $G_{P_1,P_2}^{\dagger }$: We know that $G_{P_1,P_2}$ is a unitary and so $G_{P_1,P_2}^{-1}=G_{P_1,P_2}^{\dagger }$. And the channel representation of $G_{P_1,P_2}^{\dagger }$ can be obtained by flipping some of the off-diagonal entries, depending upon the commutation relations between $P_1,P_2$ and the Paulis indexing the corresponding row and column. More details can be found in Appendix D.1.

### Multiplication by $\widehat{G_{P_1,P_2}}$

Let $U_p=\widehat{G_{P_1,P_2}}U$, where *U* is another matrix of dimension $2^{2n}\times 2^{2n}$. Then,

$$\begin{aligned} U_p[r,j] = \sum _{k=1}^{2^{2n}} \widehat{G_{P_1,P_2}}[r,k] U[k,j] \end{aligned}$$

and it can be computed very efficiently with the following observations.

1. Suppose $\widehat{G_{P_1,P_2}}[r,r]=1$. From Theorem 4.2, $\widehat{G_{P_1,P_2}}[r,s]=0$ for each $s\ne r$. Then,
  $$\begin{aligned} U_p[r,j]=\widehat{G_{P_1,P_2}}[r,r]U[r,j]=U[r,j] \qquad [\forall j\in \{1,\ldots ,2^{2n}\} ] \end{aligned}$$
  and so $U_p[r,.]\leftarrow U[r,.]$ i.e. the $r^{th}$ row of *U* gets copied into the $r^{th}$ row of $U_p$.
2. Let $\widehat{G_{P_1,P_2}}[r,r]=\frac{1}{2}$. From Theorem 4.2, we know there are 3 other non-zero off-diagonal elements. Let $\widehat{G_{P_1,P_2}}[r,s]=\pm \frac{1}{2}$, $\widehat{G_{P_1,P_2}}[r,s']=\pm \frac{1}{2}$ and $\widehat{G_{P_1,P_2}}[r,s'']=\pm \frac{1}{2}$.
  $$\begin{aligned} U_p[r,j]= & {} \widehat{G_{P_1,P_2}}[r,r]U[r,j]+\widehat{G_{P_1,P_2}}[r,s]U[s,j]+\widehat{G_{P_1,P_2}}[r,s']U[s',j]+\widehat{G_{P_1,P_2}}[r,s'']U[s'',j]\\= & {} \frac{1}{2}\left( U[r,j]\pm U[s,j]\pm U[s',j]\pm U[s'',j] \right) \end{aligned}$$
  Thus we see that the $r^{th}$ row of $U_p$ is a linear combination of the $r^{th}, s^{th}, s'^{th}, s''^{th}$ rows of *U*, multiplied by $\frac{1}{2}$, i.e. $U_p[r,.]\leftarrow \frac{1}{2}\left( U[r,.]\pm U[s,.]\pm U[s',.]\pm U[s'',.] \right)$.

Since $\frac{1}{4}^{th}$ of the diagonal in $\widehat{G_{P_1,P_2}}$ is 1 (Theorem 4.2), so we get the following result.

#### Theorem 4.9

$U_p=\widehat{G_{P_1,P_2}}U$ can be computed in time $O\left( 3\cdot 2^{4n-2} \right)$.

Currently the fastest algorithm to multiply two $2^{2n}\times 2^{2n}$ matrices has a time complexity of $2^{4.7457278n}$^53^. This modest asymptotic improvement in the complexity has a significant impact on the actual running time, especially when many such matrix multiplications are performed, as in our algorithms for exactly implementable unitaries. Also, using the representations discussed earlier, we only work with integers. This is not only faster, but also overcomes other floating point arithmetic issues like precision, etc.

$\text {sde}_2$ *of product matrix : * Let $U_p=\widehat{G_{P_1,P_2}}\widehat{U}$, where $\widehat{U}=\left( \prod _j\widehat{G_{P_{1j},P_{2j}}}\right) \widehat{C}$ is an exactly implementable unitary and $P_1,P_2,P_{1j},P_{2j}\in {\mathcal {P}}_n$, $C\in {\mathcal {C}}_n$ and $[P_1,P_2]=[P_{1j},P_{2j}]=0$. From Lemma 4.4, $\widehat{U}[k,\ell ]\in {\mathbb {Z}}\left[ \frac{1}{2}\right]$, where $k,\ell \in \{1,\ldots ,2^{2n}\}$. Thus entries of $U_p$ are of the form $\frac{1}{2}\left( v_1\pm v_2\pm v_3\pm v_4\right)$, where $v_1,v_2,v_3,v_4\in {\mathbb {Z}}\left[ \frac{1}{2}\right]$. Applying Fact 4.8 we conclude that the resulting $\text {sde}_2$ can increase by 1, remain unchanged or decrease.

We know that when we multiply by inverse then the change in sde should be reversed. Thus sde can decrease by at most 1 because it can increase by at most 1.

#### Lemma 4.10

Let $U_p=\widehat{G_{P_1,P_2}}\widehat{U}$, where $\widehat{U}=\left( \prod _j\widehat{G_{P_{1j},P_{2j} } }\right) \widehat{C}$ and $P_1,P_2,P_{1j},P_{2j}\in {\mathcal {P}}_n$, $C\in {\mathcal {C}}_n$. Then $\text {sde}_2(U_p)-\text {sde}_2(\widehat{U})\in \{\pm 1,0\}$.

## Method

In this section we describe algorithms for CS-count-optimal synthesis of multi-qubit unitaries, both exactly and approximately implementable by Clifford+CS gate set.

### CS-count-optimal synthesis algorithm for approximately implementable unitaries

Now we describe an algorithm for determining the $\epsilon$-CS-count of an *n*-qubit unitary *W*. Basically we use the procedure in^15^, but with the generating set ${\mathcal {G}}_{CS}$. This algorithm has space and time complexity $O\left( |{\mathcal {G}}_{CS}|\right)$ and $O\left( |{\mathcal {G}}_{CS}|^{{\mathcal {S}}_{\epsilon }}\right)$ respectively. We briefly describe our algorithm here. More detail pseudocode can be found in Appendix E. The optimization version, APPROX-CS-OPT (Algorithm 6), that finds the optimal CS-count is an exhaustive search procedure. In each iteration it calls the decision version APPROX-CS-DECIDE (Algorithm 7) in order to decide if ${\mathcal {S}}_{\epsilon }(W)=m$, for increasing values of a variable *m*.

The main idea to solve the decision version is as follows. Suppose we have to test if ${\mathcal {S}}_{\epsilon }(W)=m$. From the definitions given in section "Preliminaries", we know that if this is true then $\exists U\in {\mathcal {J}}_n^{CS}$ such that ${\mathcal {S}}(U)=m$ and $d(U,W)\le \epsilon$. Let $U=\left( \prod _{i=m}^1U_i\right) C_0$ where $C_0\in {\mathcal {C}}_n$ and $U_i\in {\mathcal {G}}_{CS}$. Also, we can write $W=UE$, for some unitary *E*.

21 $$\begin{aligned}{} & {} d(W,U)=\sqrt{1-\frac{|\text {Tr}(W^{\dagger }U)|}{N}}\le \epsilon \implies |\text {Tr}(W^{\dagger }U)| \ge N(1-\epsilon ^2) \end{aligned}$$

22 $$\begin{aligned}{} & {} \left| \text {Tr}\left( E^{\dagger }\right) \right| =\left| \text {Tr}\left( E\right) \right| \ge N(1-\epsilon ^2) \end{aligned}$$

The above implies that $d(E,{\mathbb {I}})\le \epsilon$. We have,

$$\begin{aligned} \left| \text {Tr}\left( W^{\dagger }\left( \prod _{j=m}^1G_{P_{1j}P_{2j}}\right) \right) \right|= & {} \left| \text {Tr}\left( E^{\dagger }U^{\dagger }\left( \prod _{j=m}^1G_{P_{1j}P_{2j}}\right) \right) \right| \\= & {} \left| \text {Tr}\left( E^{\dagger }e^{-i\phi }C_0^{\dagger }\left( \prod _{j=1}^mG_{P_{1j}P_{2j}}^{\dagger }\right) \left( \prod _{j=m}^1G_{P_{1j}P_{2j}}\right) \right) \right| \\= & {} \left| \text {Tr}\left( E^{\dagger }C_0^{\dagger }\right) \right| =\left| \text {Tr}\left( EC_0\right) \right| . \end{aligned}$$

Let ${\widetilde{U}}=\prod _{i=m}^1U_i$. To test if we have guessed the correct ${\widetilde{U}}$, we perform the following tests.

*I. Amplitude test : * We use the following theorem, which says that if *E* is close to identity then distribution of absolute-value-coefficients of $EC_0$ and $C_0$ in the Pauli basis expansion, is almost similar.

#### Theorem 5.1

(^15^) Let $E\in {\mathcal {U}}_n$ be such that $\left| \text {Tr}\left( E\right) \right| \ge N\left( 1-\epsilon ^2\right)$, for some $\epsilon \ge 0$. $C_0=\sum _{P\in {\mathcal {P}}_n}r_PP$ is an *n*-qubit Clifford. If $\left| \left\{ P:r_P\ne 0\right\} \right| =M$ then

23 $$\begin{aligned} \frac{1-\epsilon ^2}{\sqrt{M}}-\sqrt{M}\sqrt{2\epsilon ^2-\epsilon ^4}\le & {} \left| \text {Tr}\left( EC_0P_1\right) /N\right| \le \frac{1}{\sqrt{M}}+\sqrt{M}\sqrt{2\epsilon ^2-\epsilon ^4}\quad [\text {if } r_{P_1}\ne 0] \end{aligned}$$

24 $$\begin{aligned} \text {and}\quad 0\le & {} \left| \text {Tr}\left( EC_0P_1\right) /N\right| \le \sqrt{M}\sqrt{2\epsilon ^2-\epsilon ^4}\quad [\text {if }r_{P_1}=0] \end{aligned}$$

In fact, we can have a more general theorem as follows.

#### Theorem 5.2

(^15^) Let $E\in {\mathcal {U}}_n$ be such that $\left| \text {Tr}\left( E\right) \right| \ge N\left( 1-\epsilon ^2\right)$, for some $\epsilon \ge 0$. $Q=\sum _{P\in {\mathcal {P}}_n}q_PP$ is an *n*-qubit unitary. Then for each $P_1\in {\mathcal {P}}_n$,

$$\begin{aligned} (1-\epsilon ^2)|q_{P_1}|-\sum _{P\in {\mathcal {P}}_n\setminus \{P_1\}}|q_P|\sqrt{2\epsilon ^2-\epsilon ^4}\le & {} \left| \text {Tr}\left( EQP_1\right) /N\right| \le |q_{P_1}|+\sum _{P\in {\mathcal {P}}_n\setminus \{P_1\}}|q_P|\sqrt{2\epsilon ^2-\epsilon ^4}. \end{aligned}$$

So, we calculate $W'=W^{\dagger }{\widetilde{U}}$ and then calculate the set ${\mathcal {S}}_c=\left\{ |\text {Tr}(W'P)/N|:P\in {\mathcal {P}}_n\right\}$, of coefficients. Then we check if we can distinguish the following two subsets ${\mathcal {S}}_0$ and ${\mathcal {S}}_1$, for some $1\le M\le N^2$. Further details have been given in Algorithm 7.

25 $$\begin{aligned} {\mathcal {S}}_1= & {} \left\{ \left| \text {Tr}\left( EC_0P_1\right) /N\right| :r_{P_1}\ne 0\right\} \end{aligned}$$

26 $$\begin{aligned} {\mathcal {S}}_0= & {} \left\{ \left| \text {Tr}\left( EC_0P_1\right) /N\right| :r_{P_1}= 0\right\} \end{aligned}$$

After passing this test we have a unitary of the form $E^{\dagger }Q$ where *Q* is a unitary with *equal* or *nearly equal* amplitudes or coefficients (absolute value) at some points and zero or nearly zero amplitudes at other points.

*II. Conjugation test : * To ensure *Q* is a Clifford i.e. $W'=E^{\dagger }C_0$ for some $C_0\in {\mathcal {C}}_n$, we perform the conjugation test (Algorithm 8) for further verification. We use the following theorem and its corollary.

#### Theorem 5.3

(^15^) Let $E,Q\in {\mathcal {U}}_n$ such that $d(E,{\mathbb {I}})\le \epsilon$. $P'\in {\mathcal {P}}_n\setminus \{{\mathbb {I}}\}$ such that $QP'Q^{\dagger }=\sum _{P}\alpha _{P}P$, where $\alpha _{P}\in {\mathbb {C}}$. Then for each $P''\in {\mathcal {P}}_n$,

$$\begin{aligned} \min \{0,|\alpha _{P''}|(1-4\epsilon ^2+2\epsilon ^4) -2\epsilon \sum _{P\ne P''}|\alpha _P| \}\le & {} \left| \text {Tr}\left( (E^{\dagger }QP'Q^{\dagger }E)P''\right) \right| /N \\\le & {} \max \{ |\alpha _{P''}|+2\epsilon \sum _{P\ne P''}|\alpha _P|, 1\}. \end{aligned}$$

#### Corollary 5.4

(^15^) Let $C_0\in {\mathcal {C}}_n$ and $P'\in {\mathcal {P}}_n$ such that $C_0P'C_0^{\dagger }={\tilde{P}}\in {\mathcal {P}}_n$. If $E\in {\mathcal {U}}_n$ such that $d(E,{\mathbb {I}})\le \epsilon$, then

$$\begin{aligned} (1-4\epsilon ^2+2\epsilon ^4)\le & {} \left| \text {Tr}\left( (E^{\dagger }C_0P'C_0^{\dagger }E)P''\right) \right| /N\le 1 \qquad \text {if } P''={\tilde{P}}; \\ 0\le & {} \left| \text {Tr}\left( (E^{\dagger }C_0P'C_0^{\dagger }E)P''\right) \right| /N\le 2\epsilon \qquad \text {else. } \end{aligned}$$

The above theorem and corollary basically says that $EC_0$ (or $E^{\dagger }C_0$) *approximately* inherits the conjugation property of $C_0$. For each $P'\in {\mathcal {P}}_n$, if we expand $C_0P'C_0^{\dagger }$ in the Pauli basis then the absolute value of the coefficients has value 1 at one point, 0 in the rest. If we expand $EC_0P'C_0^{\dagger }E^{\dagger }$ in the Pauli basis then one of the coefficients (absolute value) will be almost 1 and the rest will be almost 0. From Theorem 5.3 this pattern will not show for at least one Pauli $P'''\in {\mathcal {P}}_n$ if we have $E^{\dagger }Q$, where $Q\notin {\mathcal {C}}_n$. If we expand $EQP'''Q^{\dagger }E^{\dagger }$ or $E^{\dagger }QP'''Q^{\dagger }E$ in the Pauli basis then the *spike in the amplitudes* will be in at least two points.

#### Synthesizing CS-count-optimal circuits

From APPROX-CS-DECIDE (Algorithm 7) we can obtain a sequence $\{U_m,\ldots ,U_1\}$ of unitaries such that $U=\left( \prod _{i=m}^1U_i\right) C_0e^{i\phi }$ (for some $C_0\in {\mathcal {C}}_n$) and ${\mathcal {S}}(U)={\mathcal {S}}_{\epsilon }(W)$. We can efficiently construct circuits for each $U_i\in {\mathcal {G}}_{CS}$, as discussed earlier. In order to determine the trailing $C_0$, we observe that from Algorithm 7 we can also obtain the following information : (1) set ${\mathcal {S}}_1$, as defined in Eq. (25), (2) $M=|{\mathcal {S}}_1|$. Thus we can calculate $r=\frac{1}{\sqrt{M}}$ and from step 4 we can actually calculate the set $\widetilde{{\mathcal {S}}_1}=\left\{ (t_P,P): |t_P|=|\text {Tr}(W'P)/N|\in {\mathcal {S}}_1\right\}$. We perform the following steps.

1. Calculate $a_P=\frac{t_P}{t_{{\mathbb {I}}}}=\frac{\text {Tr}(W'P)}{\text {Tr}(W')}$, where $(t_P,P)\in \widetilde{{\mathcal {S}}_1}$ (or equivalently $|t_P|\in {\mathcal {S}}_1$). We must remember that $(t_{{\mathbb {I}}},{\mathbb {I}})\in \widetilde{{\mathcal {S}}_1}$. It has been argued in^15^ that for small enough $\epsilon$ (say $\ll \frac{1}{M}$), $a_P\approx \frac{\overline{r_P}}{\overline{r_{{\mathbb {I}}}}}$. From Fact A.3 we know that $\frac{|\overline{r_P}|}{|\overline{r_{{\mathbb {I}}}}|}=\frac{|r_P|}{|r_{{\mathbb {I}}}|}=1$. So we adjust the fractions such that their absolute value is 1. For small enough $\epsilon$ this adjustment is not much and so with a slight abuse we use the same notation for the adjusted values.
2. Select $c,d\in {\mathbb {R}}$ such that $c^2+d^2=r^2$. Let $\widetilde{r_{{\mathbb {I}}}}=c+di$. Then we claim that the Clifford $\widetilde{C_0}=\widetilde{r_{{\mathbb {I}}}}\sum _{P:r_P\ne 0}\overline{a_P}P$ is sufficient for our purpose.

It is not hard to see that $\widetilde{C_0}=e^{i\phi '}C_0$ for some $\phi '\in [0,2\pi )$. Thus if $U'=\left( \prod _{i=m}^1U_i\right) \widetilde{C_0}$, then ${\mathcal {S}}(U')={\mathcal {S}}(U)$ and $d(U',W)\le \epsilon$. After determining the Clifford, we can efficiently construct a circuit for it, for example, by using results in^54^.

#### Complexity

Now we analyze the time and space complexity of the above algorithm.

#### Time complexity

We first analyze the time complexity of ${\mathcal {A}}_{CONJ}$. The outer and inner loop at steps 2 and 6, respectively, can run at most $2^{2n}* 2^{2n}= 2^{4n}$ times, where $N=2^n$. The complexity of the inner loop is dominated by the multiplications at step 13, which takes $O(2^{2n})$ time. Thus overall complexity of this sub-routine is $O(2^{6n})$.

Now we analyze the time complexity of APPROX-CS-DECIDE, when testing for a particular CS-count *m*. The algorithm loops over all possible products of *m* unitaries $G_{P_{1_j},P_{2_j}} \in {\mathcal {G}}_{CS}$. Thus number of iterations (steps 2-14) is at most $|{\mathcal {G}}_{CS}|^m \in O\left( n^{2m} 16^{(n-2)m} \right) \in O\left( n^{2m} 2^{(4n-8)m} \right)$, from Theorem 3.3 and this is the most expensive part of this algorithm. The *m* multiplications at step 2 and the multiplications, trace and sorting at step 4 takes $O(m2^{2n}+2^{4n})$ time. The inner loop 5-13 happens $O(2^{2n})$ times. Each of the list elements is checked and so step 8 has complexity $O(2^{2n})$. Now let within the inner loop the conjugation test is called $k_1$ times. So the loop 5-13 incurs a complexity $O(k_1\cdot 2^{6n}+(2^{2n}-k_1)2^{2n})$, when $k_1>0$, else it is $O(2^{4n})$. Let *k* be the number of outer loops (steps 2-14) for which conjugation test is invoked in the inner loop 5-13 and $k_1$ is the maximum number of times this test is called within any 5-13 inner loop. Then the overall complexity of APPROX-CS-DECIDE is $O((n^{2m}2^{(4n-8)m}-k)\cdot (m2^{2n}+2^{4n})+k\cdot (m2^{2n}+2^{4n}+k_12^{6n}+(2^{2n}-k_1)2^{2n} ) )$.

The conjugation test is invoked only if a unitary passes the amplitude test. We assume that $m<2^{2n}$ and the occurrence of non-Clifford unitaries with equal amplitude is not so frequent such that $kk_1< n^{2m}2^{(4n-8)m}-k$. Thus APPROX-CS-DECIDE has a complexity of $O(n^{2m}2^{(4n-8)m})$, for one particular value of *m*. Hence, the overall algorithm APPROX-CS-OPT has a time complexity $O\left( n^{2{\mathcal {S}}_{\epsilon }(W)} 2^{(4n-8){\mathcal {S}}_{\epsilon }(W)}\right)$.

### Space complexity

The input to our algorithm is a $2^n\times 2^n$ unitary, with space complexity $O(2^{2n})$. We need to store ${\mathcal {G}}_{CS}$, either as pairs of Paulis or as matrices. It takes space $O\left( |{\mathcal {G}}_{CS}| \right) \in O\left( n^22^{4n-8}\right)$ (Theorem 3.3). All the operations in APPROX-CS-DECIDE or ${\mathcal {A}}_{CONJ}$ either involve matrix multiplications or storing at most $2^{2n}$ numbers. Hence the overall space complexity is $O\left( n^22^{4n-8}\right)$.

### CS-count-optimal synthesis algorithm for exactly implementable unitaries

In this section we describe two algorithms for synthesizing CS-count-optimal circuits of exactly implementable unitaries. For the first one we can prove a rigorous bound on the optimality as well as space and time complexity, both of whom are exponential in the CS-count. The exponentially better second algorithm has both space and time complexity polynomial in the CS-count, but the claimed optimality as well as complexity depends on some conjectures. So we refer to it as a heuristic algorithm.

#### Nested MITM

In this section we describe the nested meet-in-the-middle (MITM) framework, introduced in^32^, that can be used to synthesize CS-count-optimal circuits. It is based on the following result that can be proved with similar arguments as in Lemma 3.1 of^33^.

##### Lemma 5.5

Let $S_i\subset U(2^n)$ be the set of all unitaries implementable in CS-count *i* over the Clifford+CS gate set. Given a unitary *U*, there exists a Clifford+CS circuit of CS-count $(m_1+m_2)$ implementing *U* if and only if $S_{m_1}^{\dagger }U\bigcap S_{m_2}\ne \emptyset$.

The pseudocode of the algorithm has been given in Appendix E (Algorithm 9). We briefly describe this algorithm here. The input consists of the unitary *U*, generating set ${\mathcal {G}}_{CS}$, test CS-count *m* and $c\ge 2$ that indicates the extent of nesting or recursion we want in our meet-in-the-middle approach. If *U* is of CS-count at most *m* then the output consists of a decomposition of *U* into smaller CS-count unitaries, else the algorithm indicates that *U* has CS-count more than *m*.

The algorithm consists of $\lceil \frac{m}{c}\rceil$ iterations and in the $i^{th}$ such iteration we generate circuits of depth *i* ($S_{i}$) by extending the circuits of depth $i-1$ ($S_{i-1}$) by one more level. Then we use these two sets to search for circuits of depth at most *ci*. In order to increase the efficiency of the search procedure we define the following. Let $\widehat{{\mathcal {J}}_n^{CS}} = \{\widehat{W}: W\in {\mathcal {J}}_n^{CS} \}$.

##### Definition 5.6

(*Coset label*^31^) Let $\widehat{W}\in \widehat{{\mathcal {J}}_n^{CS}}$. Its coset label $\widehat{W}^{(co)}$ is the matrix obtained by the following procedure. (1) Rewrite $\widehat{W}$ so that each nonzero entry has a common denominator, equal to $2^{\text {sde}(\widehat{W})}$. (2) For each column of $\widehat{W}$, look at the first non-zero entry (from top to bottom) which we write as $v=\frac{a}{2^{\text {sde}(\widehat{W})}}$. If $a<0$, multiply every element of the column by $-1$. Otherwise, if $a>0$, do nothing and move on to the next column. (3) After performing this step on all columns, permute the columns so that they are ordered lexicographically from left to right.

The following result, analogous to Proposition 2 in^31^, shows that this is a kind of equivalence function.

##### Theorem 5.7

Let $\widehat{W},\widehat{V}\in \widehat{{\mathcal {J}}_n^{CS}}$. Then $\widehat{W}^{(co)}=\widehat{V}^{(co)}$ if and only if $\widehat{W}=\widehat{V}\widehat{C}$ for some $C\in {\mathcal {C}}_n$.

In our algorithm the search is performed iteratively where in the $k^{th}$ ($1\le k\le c-1$) round we generate unitaries of CS-count at most *ki* by taking *k* unitaries $\widehat{W_1},\widehat{W_2},\ldots ,\widehat{W_k}$ where $\widehat{W_i}\in S_i$ or $\widehat{W_i}\in S_{i-1}$. Let $\widehat{W}=\widehat{W_1}\widehat{W_2}\ldots \widehat{W_k}$ and its CS-count is $k'\le ki$. We search for a unitary $\widehat{W'}$ in $S_i$ or $S_{i-1}$ such that $\left( \widehat{W}^{\dagger }\widehat{U}\right) ^{(co)}=\widehat{W'}$. By Lemma 5.5 if we find such a unitary it would imply that CS-count of *U* is $k'+i$ or $k'+i-1$ respectively. In the other direction if the CS-count of *U* is either $k'+i$ or $k'+i-1$ then there should exist such a unitary $\widehat{W'}$ in $S_i$ or $S_{i-1}$ respectively. Thus if the CS-count of *U* is at most *m* then the algorithm terminates in one such iteration and returns a decomposition of *U*. This proves the correctness of this algorithm.

*Time and space complexity* With the concept of coset label we can impose a strict lexicographic ordering on unitaries such that a set $S_i$ can be sorted with respect to this ordering in $O\left( |S_i|\log |S_i|\right)$ time and we can search for an element in this set in $O\left( \log |S_i|\right)$ time. Now consider the $k^{th}$ round of the $i^{th}$ iteration (steps 2-15 of Algorithm 9 in Appendix E). We build unitaries $\widehat{W}$ of CS-count at most *ki* using elements from $S_i$ or $S_{i-1}$. Number of such unitaries is at most $|S_i|^{k}$. Given a $\widehat{W}$, time taken to search for $W'$ in $S_i$ or $S_{i-1}$ such that $\left( \widehat{W}^{\dagger }\widehat{U}\right) ^{(co)}=\widehat{W'}$ is $O\left( \log |S_i|\right)$. Since $|S_j|\le |{\mathcal {G}}_{CS}|^j$, so the $k^{th}$ iteration of the for loop within the $i^{th}$ iteration of the while loop, takes time $O\left( |{\mathcal {G}}_{CS}|^{(c-1)i}\log |{\mathcal {G}}_{CS}| \right)$. Thus the time taken by the algorithm is $O\left( |{\mathcal {G}}_{CS}|^{(c-1)\lceil \frac{m}{c}\rceil } \log |{\mathcal {G}}_{CS}| \right)$.

In the algorithm we store unitaries of depth at most $\lceil \frac{m}{c}\rceil$. So the space complexity of the algorithm is $O\left( |{\mathcal {G}}_{CS}|^{\lceil \frac{m}{c}\rceil } \right)$. Since $|{\mathcal {G}}_{CS}|\in O\left( n^22^{4n-8} \right)$ (Theorem 3.3), so we have an algorithm with space complexity $O\left( n^{2\lceil \frac{m}{c}\rceil }2^{(4n-8)\lceil \frac{m}{c}\rceil } \right)$ and time complexity $O\left( n^{2(c-1)\lceil \frac{m}{c}\rceil }2^{(4n-8)(c-1)\lceil \frac{m}{c}\rceil } \right)$.

#### A polynomial complexity heuristic algorithm

In this section we describe a heuristic algorithm that returns a CS-count-optimal circuit in polynomial time and space. Our algorithm is motivated by the polynomial complexity T-count-optimal-synthesis algorithm in^32^. The conjectured exponential advantage comes from an efficient way of pruning the search space. The intermediate unitaries are grouped according to some properties and then we select one such group according to some criteria.

In the previous provable nested meet-in-the-middle algorithm we search for a set of $\widehat{G_{P_{1j}P_{2j}}}$ such that $\widehat{U}^{\dag }\prod _{j={\mathcal {S}}(U)}^1\widehat{G_{P_{1j}P_{2j}} }$ is $\widehat{C_0}$ for some $C_0\in {\mathcal {C}}_n$. Alternatively, we can also search for a set of $\widehat{G_{P_{1j}P_{2j}} }^{-1}$ such that $\left( \prod _{j=1}^{{\mathcal {S}}(U)}\widehat{G_{P_{1j}P_{2j}} }^{-1}\right) \widehat{U}$ is $\widehat{C_0}$ for some $C_0\in {\mathcal {C}}_n$, which is the approach taken by our heuristic algorithm. The optimization version EXACT-CS-OPT (Algorithm 10 in Appendix E) is solved by repeatedly calling EXACT-CS-DECIDE (Algorithm 11 in Appendix E), the decision version which tests whether the CS-count of an input unitary is at most an integer *m* .

**Figure 1:**
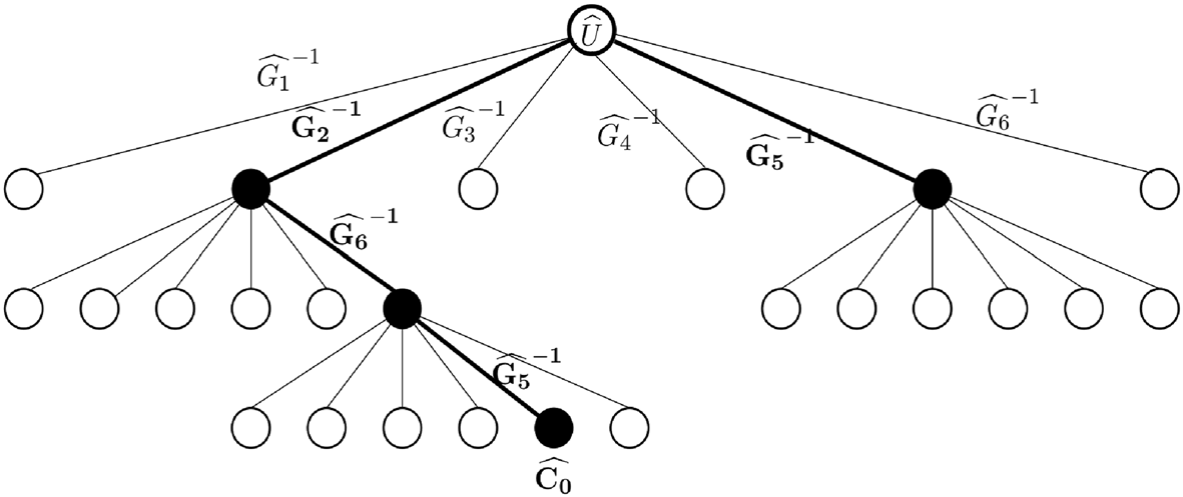
The tree built in the heuristic procedure. Each node represents a unitary and each edge represents multiplication by $\widehat{G_j}^{-1}$, where $G_j\in {\mathcal {G}}_{CS}$ is a generating set unitary. For simplicity, here we assume that there are six unitaries in ${\mathcal {G}}_{CS}$. At each level we select a set of nodes according to some changes in the properties of the child unitaries with respect to their parents. Unitaries in the next level are generated from the selected set (black nodes). We stop building the tree as soon as we reach a Clifford (here $C_0$). The path length to this node (in this case 3) is the CS-count of the unitary *U*. It also gives us a CS-count-optimal decomposition of *U*. We have $\widehat{U}=\widehat{G_2}\widehat{G_6}\widehat{G_5}\widehat{C_0}$.

It will be useful to compare the procedure EXACT-CS-DECIDE with building a tree, whose nodes store some unitary and the edges represent $\widehat{G_{P_1P_2} }^{-1}$. Thus the unitary in a child node is obtained by multiplying the unitary of the parent node with the $\widehat{G_{P_1P_2} }^{-1}$ represented by the edge. Number of children nodes of any parent node is at most $|{\mathcal {G}}_{CS}|-1$. The root stores $\widehat{U}$ and we assume it is at depth 0 (Fig. 1). We stop building this tree the moment we reach a node which stores $\widehat{C_0}$ for some $C_0\in {\mathcal {C}}_n$. This implies that the path from the root to this leaf gives a decomposition of $\widehat{U}$. In these kinds of searches the size of the tree is one of the important factors that determine the complexity of the algorithm. To reduce the complexity we try to prune this tree (Fig. 1).

At each level we try to group the nodes according to some “properties” or “parameters” of the unitaries stored in them. We hope that these parameters will “distinguish” the “correct” nodes at each level or depth of the tree and thus we will get a decomposition. Note there can be more than one decomposition of $\widehat{U}$ with the same or different CS-count. By “correct” nodes we mean those that occur in a CS-count-optimal decomposition of $\widehat{U}$. If the parameters always select only the correct nodes then we expect to get much fewer nodes at each level of the tree and the number of levels we have to build is ${\mathcal {S}}(U)$. But the parameters we select do not always distinguish the correct nodes and there are some false positives. In order for the algorithm to succeed we have to be careful so that we do not lose all correct nodes at any level and to make it efficient we have to ensure that the number of false positives are not too large and they eventually get eliminated.

We select two parameters - sde and Hamming weight of the unitaries. We know from Lemma 4.10 that sde of a child node unitary can differ by at most 1 from its parent node unitary. While building a unitary we start with $\widehat{{\mathbb {I}}_n}$ and multiply by subsequent $\widehat{G_{P_{1j}P_{2j}} }$ till we reach $\widehat{U}$. We have observed that in most of these multiplications the sde increases by 1 and the Hamming weight also gradually increases until it (Hamming weight) reaches the maximum. So while doing the inverse operations i.e. decomposing $\widehat{U}$ we expect that in most of the steps sde will decrease by 1 and as we get close to the identity, the Hamming weight will also likely decrease. If we multiply by a “wrong” $\widehat{G_{P_{1j}P_{2j}} }^{-1}$ we expect to see the same changes with much less probability, which is the probability of the false positives. This helps us distinguish the “correct” and “wrong” nodes.

Specifically, at each level we divide the set *S* of nodes into some subsets and select one of them. Below are two possible ways to divide the nodes that we have found effective. Suppose in one instance of EXACT-CS-DECIDE, *m* is the target depth (maximum depth of the tree to be built) and we have built the tree till depth *i*.

*Divide-and-select rules:*

A We divide into two sets - $S_0$ (sde increase) and $S_1$ (sde non-increase). We select the set with the minimum cardinality such that the sde of the unitaries in this set is at most $m-i$.
B. We divide into 9 sets - $S_{00}$ (both sde and Hamming weight increase), $S_{01}$ (sde increase but Hamming weight decrease), $S_{02}$ (sde increase but Hamming weight same), $S_{10}$ (sde decrease, Hamming weight increase) and $S_{11}$ (both sde and Hamming weight decrease), $S_{12}$ (sde decrease but Hamming weight same), $S_{20}$ (sde same but Hamming weight increase), $S_{21}$ (sde same but Hamming weight decrease), $S_{22}$ (both sde and Hamming weight same). We select the set with the minimum cardinality such that the sde of the unitaries in this set is at most $m-i$.

We follow any one of the above methods of divide-and-select throughout the algorithm. We note that in each of the above methods, if the cardinality of the unitaries in the selected set is more than $m-i$ then it implies we cannot get sde 0 nodes within the next few levels.

*Space and time complexity* The analysis of space and time complexity of the algorithm EXACT-CS-OPT is based on the following assumptions.

##### Conjecture 5.8

The cardinality of the set ${\widetilde{U}}$ in each iteration of EXACT-CS-DECIDE is at most $\text {poly}(n^22^{64n-8})$, when method A of divide-and-select is applied. We get at least one CS-count-optimal decomposition.

Consider the sub-routine EXACT-CS-DECIDE. There are $|{\mathcal {G}}_{CS}|-1$ multiplications by $\widehat{G_{P_{1_j},P_{2_j}} }^{-1}$ in each iteration for each unitary in ${\widetilde{U}}$. And by the above conjecture $|{\widetilde{U}}|\in \text {poly}(n^22^{4n-8})$. Thus both the time and space complexity of EXACT-CS-DECIDE are in $\text {poly}(n^22^{4n-8},m)$. We call EXACT-CS-DECIDE at most ${\mathcal {S}}(U)$ times to solve EXACT-CS-OPT. Thus space and time complexity of EXACT-CS-OPT are in $\text {poly}(n^22^{4n-8},{\mathcal {S}}(U))$.

### Implementation results

We implemented our heuristic algorithm EXACT-CS-OPT in Python on a machine with Intel(R) Core(TM) i7-5500K CPU at 2.4GHz, having 8 GB RAM and running Windows 10. The complexity of the two other algorithms described in section "Method" is exponential in number of qubits as well as CS-count. So they become intractable on a personal laptop, running on 1 core.

To test the heuristic algorithm we generate a number of random 2-qubit unitaries with CS-count at most 5, 10, 15, 20, 30 and 3-qubit unitaries with CS-count at most 5. The procedure for generating the random unitaries has been explained in Appendix E (RANDOM-CHAN-REP, Algorithm 12). We find that using Rule A, the output CS-count is at most the input CS-count for all the cases, but the time consumed is much higher. In fact, the space utilized also becomes too high. Using Rule B, the output is at most the input for all the cases for CS-count upto 10 and nearly 80–90% of the cases for CS-count more than 10, but the time consumed is much less. Here, we mention that we terminate the algorithm if it runs for more than 1 h. In Table 3 we have given the implementation results. In the last two columns we have mentioned the maximum number of unitaries that need to be stored at any point while executing the algorithm. This can be regarded as an indication of space complexity. We could not apply Rule A on 2-qubit unitaries with more than 10 CS-count or 3-qubit unitaries because it soon runs out of space. The average is computed over 10 input unitaries for each group. These include only those whose implementation was not aborted due to time being more than 1 h or space consumption being too high.

**Figure 2:**
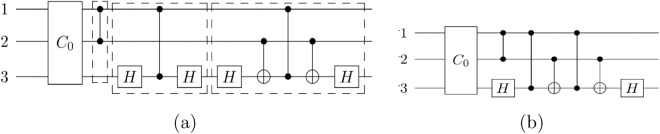
An implementation of Toffoli with 3 CS gates. $C_0$ is a 3-qubit Clifford. (**a**) The boxed parts implement the three generating set unitaries. The first box implements $G_{{\mathbb {I}}{\mathbb {I}}\text {Z},{\mathbb {I}}\text {Z}{\mathbb {I}}}$, the second one implements $G_{{\mathbb {I}}{\mathbb {I}}\text {Z},\text {X}{\mathbb {I}}{\mathbb {I}}}$ and the third box implements $G_{{\mathbb {I}}{\mathbb {I}}\text {Z},\text {X}\text {Z}{\mathbb {I}}}$. (**b**) The same circuit with some reduction of H gate.

We also implemented Toffoli (Eq. 20) using both the rules and found that its CS-count is 3. It took roughly 39 secs to get a decomposition and maximum number of unitaries stored is at most 14. For this particular case we did an exhaustive search in order to find a lower CS-count decomposition. But we found that this is the optimal CS-count for Toffoli gate. A circuit implementing Toffoli has been shown in Fig. 2. More specifically, using our heuristic algorithm EXACT-CS-OPT, using both the rules we obtain,

$$\begin{aligned} \widehat{Toffoli} = \left( \prod _{j=1}^3 \widehat{G_{P_{1_j},P_{2_j}} } \right) \widehat{C_0},\qquad [C_0\in {\mathcal {C}}_3] \end{aligned}$$

where $(P_{1_1},P_{2_1}) = ({\mathbb {I}}{\mathbb {I}}\text {Z},{\mathbb {I}}\text {Z}{\mathbb {I}})$, $(P_{1_2},P_{2_2}) = ({\mathbb {I}}{\mathbb {I}}\text {Z},\text {X}{\mathbb {I}}{\mathbb {I}})$ and $(P_{1_3},P_{2_3}) = ({\mathbb {I}}{\mathbb {I}}\text {Z},\text {X}\text {Z}{\mathbb {I}})$. It is straightforward to verify the above equation. As discussed in section "Generating set", for each generating set element $G_{P_{1_j},P_{2_j}}$ we find conjugating Cliffords $C_j\in {\mathcal {C}}_3$ such that $C_j(\text {Z}_{(a)} )C_j^{\dagger } = P_{1_j}$ and $C_j(\text {Z}_{(b)} )C_j^{\dagger } = P_{2_j}$, where $1\le a,b\le 3$ and $a\ne b$. Now $\text {H}\text {X}\text {H}=\text {Z}$ and $\text {CNOT}_{(j;k)}\text {Z}_{(k)}\text {CNOT}_{(j;k)}=\text {Z}_{(j)}\text {Z}_{(k)}$, so $(\text {H}{\mathbb {I}}{\mathbb {I}})(\text {Z}{\mathbb {I}}{\mathbb {I}})(\text {H}{\mathbb {I}}{\mathbb {I}})=\text {X}{\mathbb {I}}{\mathbb {I}}$ and $(\text {H}\cdot \text {CNOT}_{(2;3)})(\text {Z}{\mathbb {I}}{\mathbb {I}})(\text {CNOT}_{(2;3)}\cdot \text {H})=\text {X}\text {Z}{\mathbb {I}}$. Thus, we obtain the circuit as shown in Fig. 2. We have boxed the corresponding parts of the circuit that implement each generating set unitary.

**Table 3:** Implementation results of EXACT-CS-OPT on random 2 and 3-qubit unitaries

# Qubits	Input count	Avg. time (A) (s)	Avg time. (B) (s)	Max #unitaries (A)	Max #unitaries (B)
2	5	11.97	0.677	144	16
	10	163.58	1.384	1546	21
	15	–	3.25	–	26
	20	–	7.6	–	103
	30	–	10.93	–	110
3	5	–	56.23	–	12

In the second column we have the number of CS gates in the input unitary. The third and fourth column gives the average time using Divide-and-Select Rule A and B respectively. The last two columns give the maximum number of unitaries that need to be stored using rules A and B respectively

## Discussions

In this paper we have developed some theoretical concepts to better understand the unitaries implementable by the Clifford+CS gate set. First is the generating set with which we can represent any unitary exactly or approximately implementable by this gate set. We derive a bound on the cardinality of this generating set. Second is the analysis of the channel representation of these generating set elements. With the help of these tools we are able to derive some interesting results like a bound on the CS-count of arbitrary unitaries, impossibility of the exact implementation of T gate, though we know that CS can be exactly implemented by the Clifford+T gate set. This clearly shows that the set of unitaries exactly implementable by these gate sets is different. Such conclusions will help in resource estimates in various applications.

With these theoretical concepts we develop CS-count-optimal synthesis algorithms for arbitrary multi-qubit unitaries. The algorithm in^26^, that works with SO(6) representation is much faster compared to our algorithms for exactly implementable unitaries, but it only works for the 2-qubit case. Let us call this the “minimum size” case because the CS operator acts on 2 qubits. So any operator implemented by the Clifford+CS gate set acts on at least 2 qubits. The main factor behind the efficiency of this algorithm is the identification of a monotonic function or parameter in the SO(6) representation. Specifically, the authors define a parameter, “least denominator exponent” (lde), that increases with the CS-count of 2-qubit unitaries, implying the CS-count of 2-qubit unitaries is equal to the lde of its SO(6) representation. Such monotonic properties have also been identified in case of 1-qubit Clifford+T operators^31,32^. Here 1-qubit is the “minimum size” case. The T-count of 1-qubit unitaries is equal to the sde of its channel representation. Thus for the 1-qubit case it is possible to have a T-count-optimal synthesis algorithm with complexity linearly dependent on the T-count. But this monotonicity does not hold for multi-qubit case or “non-minimum” size case of Clifford+T operators^32^ and so it becomes challenging to design efficient algorithms for the multi-qubit case. Similar monotonic properties have also been identified in case of 1-qubit (“minimum size”) Clifford+V operators^9^, but we show in a separate paper^41^ that it does not hold for the multi-qubit case. For Clifford+CS operators we are not aware of any such monotonic property in the channel representation, even for the 2-qubit “minimum size” case. We do not know about the difficulty of generalizing the $SU(4) \mapsto SO(6)$ mapping in^26^ to $SU(2^n)$. And even if it is done, then whether the stated monotonic property holds or breaks down as in the case of Clifford+T and Clifford+V operators. These maybe some interesting but challenging directions for future research.

The theory and techniques developed in this paper have a much broader scope and can be applied to study other universal gate sets and design more efficient algorithms, possibly by sacrificing the optimality criterion. These concepts can also be used to design CS-depth-optimal quantum circuits, analogous to the work in^33^ for T-depth-optimality. We leave these for future research.

### Supplementary Information

Supplementary Information.

## Data Availability

All codes can be found online at the public repository https://github.com/PriyankaMukhopadhyay/CS-opt.
