# Supplementary material for: CS-count-optimal quantum circuits for arbitrary multi-qubit unitaries
Source: Sci Rep. 2024 Jun 17;14:13916. doi: 10.1038/s41598-024-64558-8 (PMC11183131; doi:10.1038/s41598-024-64558-8)
Supplement: Supplementary file 1 — Supplementary Information. [file 41598_2024_64558_MOESM1_ESM.pdf]

Supplementary Information : CS-count-optimal quantum circuits for arbitrary multi-qubit unitaries

Priyanka Mukhopadhyay *[1]

[1]Department of Computer Science, University of Toronto, ON, Canada

A Some additional preliminaries

A.1 Cliffords and Paulis

The *single qubit Pauli matrices* are as follows:

$$\mathrm{X} = \begin{bmatrix} 0 & 1 \\ 1 & 0 \end{bmatrix} \qquad \mathrm{Y} = \begin{bmatrix} 0 & -i \\ i & 0 \end{bmatrix} \qquad \mathrm{Z} = \begin{bmatrix} 1 & 0 \\ 0 & -1 \end{bmatrix}$$

Parenthesized subscripts are used to indicate qubits on which an operator acts For example, $\mathrm{X}_{(1)} = \mathrm{X} \otimes \mathbb{I}^{\otimes(n-1)}$ implies that Pauli X matrix acts on the first qubit and the remaining qubits are unchanged.

The *n-qubit Pauli operators* are : $\mathcal{P}_n = \{Q_1 \otimes Q_2 \otimes \ldots \otimes Q_n : Q_i \in \{\mathbb{I}, \mathrm{X}, \mathrm{Y}, \mathrm{Z}\}\}$.

The *single-qubit Clifford group* $\mathcal{C}_1$ is generated by the Hadamard and phase gates : $\mathcal{C}_1 = \langle \mathrm{H}, \mathrm{S} \rangle$ where

$$\mathrm{H} = \frac{1}{\sqrt{2}} \begin{bmatrix} 1 & 1 \\ 1 & -1 \end{bmatrix} \qquad \mathrm{S} = \begin{bmatrix} 1 & 0 \\ 0 & i \end{bmatrix}$$

When $n > 1$ the *n-qubit Clifford group* $\mathcal{C}_n$ is generated by these two gates (acting on any of the n qubits) along with the two-qubit $\mathrm{CNOT} = |0\rangle\langle 0| \otimes \mathbb{I} + |1\rangle\langle 1| \otimes \mathrm{X}$ gate (acting on any pair of qubits).

The Clifford group is special because of its relationship to the set of n-qubit Pauli operators. Cliffords map Paulis to Paulis, up to a possible phase of -1, i.e. for any $P \in \mathcal{P}_n$ and any $C \in \mathcal{C}_n$ we have $CPC^\dagger = (-1)^b P'$ for some $b \in \{0, 1\}$ and $P' \in \mathcal{P}_n$. In fact, given two Paulis (neither equal to the identity), it is always possible to efficiently find a Clifford which maps one to the other.

Fact A.1 ([1])**.** For any $P, P' \in \mathcal{P}_n \setminus \{\mathbb{I}\}$ there exists a Clifford $C \in \mathcal{C}_n$ such that $CPC^\dagger = P'$. A circuit for C over the gate set $\{\mathrm{H}, \mathrm{S}, \mathrm{CNOT}\}$ can be computed efficiently (as a function of n).

Fact A.2 ([2])**.** Let $Q = \sum_{P \in \mathcal{P}_n} q_P P$ be the expansion of a matrix Q in the Pauli basis. Then

$$q_P = \mathrm{Tr}\,(QP)\,/N \qquad [N = 2^n].$$

*mukhopadhyay.priyanka@gmail.com, priyanka.mukhopadhyay@utoronto.ca

Further, if Q is a unitary then

$$\sum_{P\in\mathcal{P}_n} |q_P|^2 = 1$$

We observe the following when expanding a Clifford in the Pauli basis.

Fact A.3 ([3])**.** If $C \in \mathcal{C}_n$ then for each $P \in \mathcal{P}_n$ $\exists r_P \in \mathbb{C}$, such that $C = \sum_{P\in\mathcal{P}_n} r_P P$. Further, if $r_P, r_{P'} \neq 0$ for any pair of P, P', then $|r_P| = |r_{P'}| = r$, for some $r \in \mathbb{R}$.

Fact A.4. If $[A, B] = 0$ and $CAC^\dagger = A_1$, $CBC^\dagger = B_1$, then $[A_1, B_1] = 0$.

Proof. We have the following.

$$\begin{aligned} A_1B_1 &= (CAC^\dagger)(CBC^\dagger) = C(AB)C^\dagger = C(BA)C^\dagger \\ &= (CBC^\dagger)(CAC^\dagger) = B_1A_1 \end{aligned}$$

□

B Generating set $\mathcal{G}_{CS}$

Lemma B.1. *If $P_1, P_2 \in \mathcal{P}_n \setminus \{\mathbb{I}\}$ such that $[P_1, P_2] = 0$, then*

1. $G_{P_1,P_2} = G_{P_2,P_1}$*;*
2. $G_{P_1,-P_1P_2} = G_{P_1,P_2}$*;*
3. $G_{P_1,-P_2} = G_{P_1,P_2}C$*, where $C \in \mathcal{C}_n$ is an n-qubit Clifford.*

Proof. (1) is easy to see. Even (2) is straight-forward because

$$\begin{aligned} G_{P_1,-P_1P_2} &= \beta_0\mathbb{I} + \beta_1(P_1 - P_1P_2 - P_1(-P_1P_2)) \\ &= \beta_0\mathbb{I} + \beta_1(P_1 + P_2 - P_1P_2) = G_{P_1,P_2} \end{aligned}$$

Now we prove (3). Since $CS_{(i,j)} = \exp\left(\frac{i\pi}{8}(\mathbb{I} - \mathrm{Z}_{(i)} - Z_{(j)} + \mathrm{Z}_{(i)}\mathrm{Z}_{(j)})\right)$, so we can alternatively write G_{P_1,P_2} as follows.

$$\begin{aligned} G_{P_1,P_2} &= C(CS_{(i,j)})C^\dagger = \exp\left(\frac{i\pi}{8}(\mathbb{I} - P_1 - P_2 + P_1P_2)\right) \\ G_{P_1,-P_2} &= \exp\left(\frac{i\pi}{8}(\mathbb{I} - P_1 + P_2 - P_1P_2)\right) \\ &= \exp\left(\frac{i\pi}{8}(\mathbb{I} - P_1 - P_2 + P_1P_2) + \frac{i\pi}{8}(2P_2 - 2P_1P_2)\right) \\ &= G_{P_1,P_2}\exp\left(\frac{i\pi}{4}P_2\right)\exp\left(-\frac{i\pi}{4}P_1P_2\right) \qquad (1) \end{aligned}$$

We prove that $\exp\left(\frac{i\pi}{4}P_2\right)$ and $\exp\left(-\frac{i\pi}{4}P_1P_2\right)$ are Cliffords. Let $B \in \mathcal{P}_n$. Then,

$$\begin{aligned}
\exp(\frac{i\pi}{4}P_2)B\exp(\frac{i\pi}{4}P_2)^\dagger &= \frac{\mathbb{I}+iP_2}{\sqrt{2}}B\frac{\mathbb{I}-iP_2}{\sqrt{2}} \\
&= P_2\left(\frac{P_2B+BP_2}{2}\right)+i\left(\frac{P_2B-BP_2}{2}\right) \\
&= B \quad \text{if } P_2B=BP_2; \quad iB \quad \text{if } P_2B=-BP_2
\end{aligned}$$

Since B is a Pauli, so iP_2B is a Pauli and this implies that $\exp\left(\frac{i\pi}{4}P_2\right) \in \mathcal{C}_n$. Similarly, $\exp\left(-\frac{i\pi}{4}P_1P_2\right) \in \mathcal{C}_n$. Thus, (3) is proved from Equation 1.

□

We know that $\mathcal{G}_{CS}$ is the set of unitaries $G_{P_1,P_2} = \left(\frac{3+i}{4}\right)\mathbb{I}_n + \left(\frac{1-i}{4}\right)(P_1+P_2-P_1P_2)$, such that

1. $P_1, P_2 \in \mathcal{P}_n \setminus \{\mathbb{I}\}$, $P_1 \neq P_2$ and $[P_1, P_2] = 0$.
2. $(P_1,P_2) \equiv (P_2,P_1) \equiv (P_1, \pm P_1P_2)$. The equivalence implies that we include only one of them in the set.

We call this a *generating set* (modulo Clifford), because any unitary exactly implementable by the Clifford+CS gate set can be written (up to a global phase) as a product of unitaries from this set and a Clifford.

Theorem B.2.

$$\begin{aligned}
|\mathcal{G}_{CS}| &\leq \frac{1}{8}(16^n - 13^n - 4^n + 1) + \frac{1}{12}(12^n - 2\cdot 6^n) \\
&\in O\left(n^2 16^{n-2}\right)
\end{aligned}$$

Proof. Let $P = \bigotimes_{j=1}^n P_j \in \mathcal{P}_n \setminus \{\mathbb{I}_n\}$ such that it is the tensor product of m non-identity single-qubit Paulis. Without loss of generality, let us assume that $P_j \neq \mathbb{I}$ when $1 \leq j \leq m$. Let S_{1Pm} is the set of non-identity Paulis that commute with P and have $\mathbb{I}$ on the first m 1-qubit subspaces i.e. $Q = \bigotimes_{j=1}^n Q_j \in S_{1Pm}$ if $Q_j = \mathbb{I}$ for $1 \leq j \leq m$. This implies $[P,Q]=0$. When $m+1 \leq j \leq n$, then $Q_j \in \mathcal{P}_1$ and so there can be 4 possible options. Excluding the condition when $Q_j = \mathbb{I}$ for all j, we have $|S_{1Pm}| = 4^{n-m}-1$.

Let S_{2Pm} is the set of non-identity Paulis that commute with P and have $\mathbb{I}$ on the last $n-m$ 1-qubit subspaces i.e. $Q' = \bigotimes_{j=1}^n Q'_j \in S_{2Pm}$ if $Q'_j = \mathbb{I}$ for $m+1 \leq j \leq n$. Here, we want to enforce the constraint that $(P,P') \equiv (P, \pm PP')$ i.e. $\nexists R \in S_{2Pm}$ such that $Q' = \pm PR$. Now, $[P,Q']=0$ if and only if $k = \left|\{j : P_j \neq Q'_j; 1 \leq j \leq m\}\right|$ is even. In the remaining $m-k$ subspaces $Q'_j = P_j$ or $\mathbb{I}$. There can be at most 2^{m-k} combinations. In the k unequal (but neither is identity) subspaces there can be at most 2^k combinations, but out of these half can be obtained by myltiplying P with the other half. For example, consider $P = \text{XX}$, then $Q' \in \{\text{YY}, \text{YZ}\}$. Though $\{\text{ZZ}, \text{ZY}\}$ also commute with P but these can be obtained by multiplying P with YY and YZ. Thus, there can be $2^{k-1}\cdot 2^{m-k} = 2^{m-1}$ possibilities for Q'. Now we can select k subspaces in $\binom{m}{k}$ ways. When $m < n$ then k can vary from 0 to m or $m-1$, whichever is even. When $m = n$ then k varies from 2 to m

or $m-1$, because we want to avoid the all identity case. Hence,

$$
\begin{aligned}
|S_{2Pm}| &\leq 2^{m-1}\sum_{k=0}^{m'}\binom{m}{k} := 2^{m-1}\cdot A \quad [m<n, m'=m \text{ or } m-1] \\
S_{2Pm}| &\leq 2^{m-1}\sum_{k=2}^{m'}\binom{m}{k} = 2^{m-1}\left(\sum_{\ell=0}^{k}\binom{m}{\ell}-1\right) := 2^{m-1}\cdot(A-1) \quad [m=n, m'=m \text{ or } m-1]
\end{aligned}
$$

When m is even then,

$$
A = \sum_{k=0}^{m}\binom{m}{k} = \frac{1}{2}\left(\sum_{i=0}^{m}\binom{m}{i}+\sum_{i=0}^{m}(-1)^i\binom{m}{i}\right) = \frac{1}{2}((1+1)^m+(1-1)^m) = 2^{m-1};
$$

while when $m-1$ is even then,

$$
A = \sum_{k=0}^{m-1}\binom{m}{k} = \frac{1}{2}\left(\sum_{i=0}^{m}\binom{m}{i}+\sum_{i=0}^{m}(-1)^i\binom{m}{i}\right) = 2^{m-1}.
$$

So,

$$
\begin{aligned}
|S_{2Pm}| &\leq 2^{m-1}2^{m-1} = 2^{2m-2} \qquad [1\leq m<n] \\
|S_{2Pm}| &\leq 2^{m-1}\left(2^{m-1}-1\right) = 2^{2m-2}-2^{m-1} \qquad [m=n]
\end{aligned} \tag{2}
$$

Let d_{Pm} is the number of Paulis that commute with P and we maintain the equivalence $(P,Q)\equiv(P,\pm PQ)$ i.e. we count either one of P and PQ. Then,

$$
\begin{aligned}
d_P &\leq |S_{1Pm}| \leq 4^{n-1}-1 \qquad [m=1] \\
&\leq |S_{2Pm}| \leq 2^{2n-2}-2^{n-1} \qquad [m=n] \\
&\leq |S_{1Pm}|\cdot|S_{2Pm}| \leq \left(2^{2n-2m}-1\right)2^{2m-2} = 2^{2n-2}-2^{2m-2} = \frac{1}{4}\left(4^n-4^m\right) \qquad [1<m<n]
\end{aligned}
$$

We count the number of Paulis $P'=\bigotimes_{j=1}^{n}P'_j$, which are tensor product of m non-identity Paulis. We denote it by d_m. There can be $\binom{n}{m}3^m$ of them. When $m=n$, among them there are few which are products of the other. Specifically we can take 2 possible values for P'_1 and 3 possible values for the remaining P'_j, where $1<j\leq n$. The remaining n-qubit Paulis that have m non-identity tensored components, can be obtained as product of these. And we do not count these, due to the equivalence constraint. When $m=1$ there can be 3 possibilities for the only non-identity component. So number of possible Paulis with m non-identity tensored components is at most $3\cdot\binom{n}{1}$ if $m=1$; $\binom{n}{n}2\cdot 3^{n-1}$ if $m=n$ and $\binom{n}{m}3^m$ if $1<m<n$. Hence,

$$
\begin{aligned}
d_m &\leq \binom{n}{1}3(4^{n-1}-1) = 3n(4^{n-1}-1) \qquad \text{when } m=1; \\
d_m &\leq \binom{n}{m}\frac{3^m}{4}\left(2^{2n}-2^{2m}\right) = \binom{n}{m}\left(4^{n-1}\cdot 3^m-\frac{1}{4}\cdot 12^m\right) \qquad \text{when } n>m\geq 2; \\
d_m &\leq \binom{n}{n}2\cdot 3^{n-1}\left(2^{2n-2}-2^{n-1}\right) = 2\left(12^{n-1}-6^{n-1}\right) \qquad \text{when } m=n.
\end{aligned}
$$

Total number of Pauli pairs is,

$$\begin{aligned}\sum_m d_m &\leq 3n(4^{n-1}-1)+2(12^{n-1}-6^{n-1})+\sum_{m=2}^{n-1}\binom{n}{m}\left(4^{n-1}\cdot 3^m-\frac{1}{4}\cdot 12^m\right)\\ &:= \frac{3n}{4}4^n-3n+\frac{12^n}{6}-\frac{6^n}{3}+B;\end{aligned}$$

where

$$\begin{aligned}B &= \frac{4^n}{4}\left[\sum_{m=0}^{n}\binom{n}{m}3^m-\binom{n}{0}-\binom{n}{1}3-\binom{n}{n}3^n\right]-\frac{1}{4}\left[\sum_{m=0}^{n}\binom{n}{m}12^m-\binom{n}{0}-\binom{n}{1}12-\binom{n}{n}12^n\right]\\ &= \frac{4^n}{4}\left[4^n-1-3n-3^n\right]-\frac{1}{4}\left[13^n-1-12n-12^n\right]=\frac{16^n}{4}-\frac{13^n}{4}-4^n\frac{3n+1}{4}+3n+\frac{1}{4};\end{aligned}$$

and so,

$$\sum_m d_m \leq \frac{16^n}{4}-\frac{13^n}{4}+\frac{12^n}{6}-\frac{6^n}{3}-\frac{4^n}{4}+\frac{1}{4}.$$

Since the number of pairs are permutation invariant i.e. $(P_1,P_2)\equiv(P_2,P_1)$, so,

$$|\mathcal{G}_{CS}|=\frac{1}{2}\sum_m d_m \leq \frac{1}{8}(16^n-13^n-4^n+1)+\frac{1}{12}(12^n-2\cdot 6^n). \tag{3}$$

We observe the fact that for any $a,b\in\mathbb{R}$ such that $a\geq b>1$ and $m\in\mathbb{Z}_+$ we have

$$a^m-b^m=(a-b)(a^{m-1}+a^{m-2}b+a^{m-3}b^2+\ldots+b^{m-1}),$$

and so

$$(a-b)mb^{m-1}\leq a^m-b^m\leq (a-b)ma^{m-1}.$$

Thus,

$$\begin{aligned}|\mathcal{G}_{CS}| &\leq \frac{1}{8}\left(3n16^{n-1}-3n1^{n-1}\right)+\frac{1}{12}\left(12^n-6^n\right)\\ &\leq \frac{3n}{8}15(n-1)16^{n-2}+\frac{6n}{12}12^{n-1}\\ &\leq \frac{45n^2}{8}16^{n-2}+\frac{6n\cdot 12}{12}12^{n-2}\leq\frac{45n^2}{8}16^{n-2}+6n16^{n-2}\\ &\leq n^2 16^{n-2}\left(\frac{45}{8}+6\right)=11.625n^2 16^{n-2},\end{aligned}$$

and hence the theorem is proved. □

C Bound on CS-count of arbitrary unitaries

In this section we derive some results required to prove a lower bound on the CS-count of arbitrary n-qubit unitaries, as discussed in Section 3.1.

Lemma C.1. *If* $\widetilde{U} = \prod_{j=1}^{m} G_{P_{1_j},P_{2_j}}$*, where* $G_{P_{1_j},P_{2_j}} = a\mathbb{I} + bQ_j$*,* $Q_j = P_{1_j} + P_{2_j} - P_{1_j}P_{2_j}$*,* $a = \frac{3+i}{4}$ *and* $b = \frac{1-i}{4}$*, then*

$$\begin{aligned}\widetilde{U} &= a^m\mathbb{I} + a^{m-1}b\left(\sum_j Q_j\right) + a^{m-2}b^2\left(\sum_{j_1<j_2} Q_{j_1}Q_{j_2}\right) + a^{m-3}b^3\left(\sum_{j_1<j_2<j_3} Q_{j_1}Q_{j_2}Q_{j_3}\right) + \cdots \\ &\quad \cdots + ab^{m-1}\left(\sum_{j_1<j_2<\cdots<j_{m-1}} Q_{j_1}Q_{j_2}\cdots Q_{j_{m-1}}\right) + b^m\prod_{j=1}^{m} Q_j\end{aligned}$$

Proof. We prove this by induction. The statement holds trivially for $m = 1$. For $m = 2$ we have

$$\prod_{j=1}^{2} G_{P_{1_j},P_{2_j}} = (a\mathbb{I} + bQ_1)(a\mathbb{I} + bQ_2) = a^2\mathbb{I} + ab(Q_1 + Q_2) + b^2Q_1Q_2.$$

We assume that the statement holds for $m - 1$, that is

$$\begin{aligned}\prod_{j=1}^{m-1} G_{P_{1_j},P_{2_j}} &= a^{m-1}\mathbb{I} + a^{m-2}b\left(\sum_{j=1}^{m-1} Q_j\right) + a^{m-3}b^2\left(\sum_{\substack{j_1,j_2=1\\ j_1<j_2}}^{m-1} Q_{j_1}Q_{j_2}\right) + \cdots \\ &\quad \cdots + ab^{m-2}\left(\sum_{\substack{j_1,j_2,\ldots,j_{m-2}=1\\ j_1<j_2<\cdots<j_{m-2}}}^{m-1} Q_{j_1}Q_{j_2}\cdots Q_{j_{m-2}}\right) + b^{m-1}\prod_{j=1}^{m-1} Q_j.\end{aligned}$$

Therefore,

$$
\begin{aligned}
\widetilde{U} &= \left(\prod_{j=1}^{m-1} G_{P_{1_j},P_{2_j}}\right)(a\mathbb{I}+bQ_m) \\
&= \left(a^{m-1}\mathbb{I} + a^{m-2}b\left(\sum_{j=1}^{m-1} Q_j\right) + a^{m-3}b^2\left(\sum_{\substack{j_1,j_2=1\\ j_1<j_2}}^{m-1} Q_{j_1}Q_{j_2}\right) + \cdots\right. \\
&\qquad \left.\cdots + ab^{m-2}\left(\sum_{\substack{j_1,j_2,\ldots,j_{m-2}=1\\ j_1<j_2<\cdots<j_{m-2}}}^{m-1} Q_{j_1}Q_{j_2}\cdots Q_{j_{m-2}}\right) + b^{m-1}\prod_{j=1}^{m-1} Q_j\right)(a\mathbb{I}+bQ_m) \\
&= a^m\mathbb{I} + a^{m-1}b\left(\sum_{j=1}^{m-1} Q_j\right) + a^{m-2}b^2\left(\sum_{\substack{j_1,j_2=1\\ j_1<j_2}}^{m-1} Q_{j_1}Q_{j_2}\right) + \cdots + a^2b^{m-2}\left(\sum_{\substack{j_1,j_2,\ldots,j_{m-2}=1\\ j_1<j_2<\cdots<j_{m-2}}}^{m-1} Q_{j_1}Q_{j_2}\cdots Q_{j_{m-2}}\right) \\
&\qquad + ab^{m-1}\prod_{j=1}^{m-1} Q_j + a^{m-1}bQ_m + a^{m-2}b^2\left(\sum_{j=1}^{m-1} Q_j\right)Q_m + a^{m-3}b^3\left(\sum{}_{\substack{j_1,j_2=1\\ j_1<j_2}}^{m-1} Q_{j_1}Q_{j_2}\right)Q_m + \cdots \\
&\qquad \cdots + ab^{m-1}\left(\sum_{\substack{j_1,j_2,\ldots,j_{m-2}=1\\ j_1<j_2<\cdots<j_{m-2}}}^{m-1} Q_{j_1}Q_{j_2}\cdots Q_{j_{m-2}}\right)Q_m + b^m\left(\prod_{j=1}^{m-1} Q_j\right)Q_m \\
&= a^m\mathbb{I} + a^{m-1}b\left(\sum_j Q_j\right) + a^{m-2}b^2\left(\sum_{j_1<j_2} Q_{j_1}Q_{j_2}\right) + a^{m-3}b^3\left(\sum_{j_1<j_2<j_3} Q_{j_1}Q_{j_2}Q_{j_3}\right) + \cdots \\
&\qquad \cdots + ab^{m-1}\left(\sum_{j_1<j_2<\cdots<j_{m-1}} Q_{j_1}Q_{j_2}\cdots Q_{j_{m-1}}\right) + b^m\prod_{j=1}^{m} Q_j,
\end{aligned}
$$

thus proving the lemma. □

C.1 Pauli basis expansion of rotation unitaries

Now we derive the Pauli basis expansions of the rotation unitaries considered in Section 3.1.1. For convenience, especially when working with more than 2-qubit unitaries, we use $\mathrm{diag}(d_1, d_2, \ldots, d_N)$ to denote an $N \times N$ diagonal matrix with entries $d_1, d_2, \ldots, d_N$ along the diagonal and 0 in the remaining places.

First we consider the single qubit z-rotation R_z gate.

$$
\begin{aligned}
R_z(\theta) &= e^{-i\frac{\theta}{2}}\begin{bmatrix}1 & 0\\ 0 & e^{i\theta}\end{bmatrix} = e^{-i\frac{\theta}{2}}\frac{1}{2}\begin{bmatrix}(1+e^{i\theta})+(1-e^{i\theta}) & 0\\ 0 & (1+e^{i\theta})-(1-e^{i\theta})\end{bmatrix} \\
&= e^{-i\frac{\theta}{2}}\frac{1}{2}\left((1+e^{i\theta})\begin{bmatrix}1 & 0\\ 0 & 1\end{bmatrix} + (1-e^{i\theta})\begin{bmatrix}1 & 0\\ 0 & -1\end{bmatrix}\right) \\
&= e^{-i\frac{\theta}{2}}\left(\frac{1+e^{i\theta}}{2}\mathbb{I} + \frac{1-e^{i\theta}}{2}\mathrm{Z}\right) \qquad (4)
\end{aligned}
$$

$R_z(\theta) = e^{-i\frac{\theta}{2}}R_n(\theta)$ i.e. these two rotation gates are equivalent upto a global phase, but not their controlled versions. Now we consider $R_n(\theta)$, controlled on 1 qubit. With similar derivation as in Equation 4, we first express

$$
cR_n(\theta) = \begin{bmatrix}1 & 0 & 0 & 0\\ 0 & 1 & 0 & 0\\ 0 & 0 & 1 & 0\\ 0 & 0 & 0 & e^{i\theta}\end{bmatrix} = \frac{1+e^{i\theta}}{2}(\mathbb{I}\otimes\mathbb{I}) + \frac{1-e^{i\theta}}{2}\mathrm{CZ}, \qquad (5)
$$

where the CZ gate can be expressed,

$$
CZ = \begin{bmatrix}1 & 0 & 0 & 0\\ 0 & 1 & 0 & 0\\ 0 & 0 & 1 & 0\\ 0 & 0 & 0 & -1\end{bmatrix} = \frac{1}{2}\left(\mathbb{I}\otimes\mathbb{I} + \mathrm{Z}\otimes\mathbb{I} + \mathbb{I}\otimes\mathrm{Z} - \mathrm{Z}\otimes\mathrm{Z}\right) \qquad (6)
$$

and thus

$$
\begin{aligned}
cR_n(\theta) &= \frac{1+e^{i\theta}}{2}(\mathbb{I}\otimes\mathbb{I}) + \frac{1-e^{i\theta}}{2}\left(\frac{1}{2}\left(\mathbb{I}\otimes\mathbb{I} + \mathrm{Z}\otimes\mathbb{I} + \mathbb{I}\otimes\mathrm{Z} - \mathrm{Z}\otimes\mathrm{Z}\right)\right) \\
&= \frac{3+e^{i\theta}}{4}(\mathbb{I}\otimes\mathbb{I}) + \frac{1-e^{i\theta}}{4}\left((\mathrm{Z}\otimes\mathbb{I}) + (\mathbb{I}\otimes\mathrm{Z}) - (\mathrm{Z}\otimes\mathrm{Z})\right). \qquad (7)
\end{aligned}
$$

Next, we consider $cR_z(\theta)$ unitary i.e. z-rotation controlled on 1 qubit.

$$
\begin{aligned}
cR_z(\theta) &= \begin{bmatrix}1 & 0 & 0 & 0\\ 0 & 1 & 0 & 0\\ 0 & 0 & e^{-i\theta/2} & 0\\ 0 & 0 & 0 & e^{i\theta/2}\end{bmatrix} \\
&= \mathrm{diag}\left(1, 1, \cos(\theta/2), \cos(\theta/2)\right) + \mathrm{diag}\left(0, 0, -i\sin(\theta/2), i\sin(\theta/2)\right) \\
&= \frac{1}{2}\left(\left(1+\cos\frac{\theta}{2}\right)\mathrm{diag}(1,1,1,1) + \left(1-\cos\frac{\theta}{2}\right)\mathrm{diag}(1,1,-1,-1)\right) \\
&\quad + \frac{i}{2}\left(\left(\sin\frac{\theta}{2}\right)\mathrm{diag}(1,-1,-1,1) - \left(\sin\frac{\theta}{2}\right)\mathrm{diag}(1,-1,1,-1)\right) \\
&= \left(\frac{1+\cos\frac{\theta}{2}}{2}\right)(\mathbb{I}\otimes\mathbb{I}) + \left(\frac{1+\cos\frac{\theta}{2}}{2}\right)(\mathrm{Z}\otimes\mathbb{I}) \\
&\quad -i\left(\frac{\sin\frac{\theta}{2}}{2}\right)(\mathbb{I}\otimes\mathrm{Z}) + i\left(\frac{\sin\frac{\theta}{2}}{2}\right)(\mathrm{Z}\otimes\mathrm{Z}) \qquad (8)
\end{aligned}
$$

Now, we consider the 2-qubit Given's rotation.

$$\begin{aligned}
Givens(\theta) &= \begin{bmatrix} 1 & 0 & 0 & 0 \\ 0 & \cos(\theta) & -\sin(\theta) & 0 \\ 0 & \sin(\theta) & \cos(\theta) & 0 \\ 0 & 0 & 0 & 1 \end{bmatrix} \\
&= \begin{bmatrix} 1 & 0 & 0 & 0 \\ 0 & \cos(\theta) & 0 & 0 \\ 0 & 0 & \cos(\theta) & 0 \\ 0 & 0 & 0 & 1 \end{bmatrix} + \begin{bmatrix} 0 & 0 & 0 & 0 \\ 0 & 0 & -\sin(\theta) & 0 \\ 0 & \sin(\theta) & 0 & 0 \\ 0 & 0 & 0 & 0 \end{bmatrix} := A + B
\end{aligned}$$

Using arguments as before, we can express A as follows.

$$\begin{aligned}
A &= \mathrm{diag}(1, \cos\theta, \cos\theta, 1) = \frac{1}{2}\left((1+\cos\theta)\mathrm{diag}(1,1,1,1) + (1-\cos\theta)\mathrm{diag}(1,-1,-1,1)\right) \\
&= \left(\frac{1+\cos\theta}{2}\right)(\mathbb{I}\otimes\mathbb{I}) + \left(\frac{1-\cos\theta}{2}\right)(\mathrm{Z}\otimes\mathrm{Z})
\end{aligned}$$

We expand B as follows.

$$\begin{aligned}
B &= \frac{1}{2i}\left(\sin\theta \begin{bmatrix} 0 & 0 & 0 & i \\ 0 & 0 & -i & 0 \\ 0 & i & 0 & 0 \\ -i & 0 & 0 & 0 \end{bmatrix} + \sin\theta \begin{bmatrix} 0 & 0 & 0 & -i \\ 0 & 0 & -i & 0 \\ 0 & i & 0 & 0 \\ i & 0 & 0 & 0 \end{bmatrix}\right) \\
&= -i\frac{\sin\theta}{2}\left(-(\mathrm{X}\otimes\mathrm{Y}) + (\mathrm{Y}\otimes\mathrm{X})\right) \qquad (9)
\end{aligned}$$

So,

$$Givens(\theta) = \left(\frac{1+\cos\theta}{2}\right)(\mathbb{I}\otimes\mathbb{I}) + i\left(\frac{\sin\theta}{2}\right)(\mathrm{X}\otimes\mathrm{Y}) - i\left(\frac{\sin\theta}{2}\right)(\mathrm{Y}\otimes\mathrm{X}) + \left(\frac{1-\cos\theta}{2}\right)(\mathrm{Z}\otimes\mathrm{Z}).$$

Now we consider the following rotation gates, controlled on 2 qubits. Similar to Equation 5,

$$ccR_n(\theta) = \mathrm{diag}\left(1,1,1,1,1,1,1,e^{i\theta}\right) = \left(\frac{1+e^{i\theta}}{2}\right)(\mathbb{I}\otimes\mathbb{I}\otimes\mathbb{I}) + \left(\frac{1+e^{i\theta}}{2}\right)CCZ,$$

where the double-controlled Z unitary can be expanded as,

$$\begin{aligned}
CCZ &= \mathrm{diag}\left(1,1,1,1,1,1,1,-1\right) = \frac{3}{4}(\mathbb{I}\otimes\mathbb{I}\otimes\mathbb{I}) + \frac{1}{4}\left((\mathbb{I}\otimes\mathbb{I}\otimes\mathrm{Z}) + (\mathbb{I}\otimes\mathrm{Z}\otimes\mathbb{I}) + (\mathrm{Z}\otimes\mathbb{I}\otimes\mathbb{I})\right) \\
&\quad -\frac{1}{4}\left((\mathbb{I}\otimes\mathrm{Z}\otimes\mathrm{Z}) + (\mathrm{Z}\otimes\mathrm{Z}\otimes\mathbb{I}) + (\mathrm{Z}\otimes\mathbb{I}\otimes\mathrm{Z})\right) + \frac{1}{4}(\mathrm{Z}\otimes\mathrm{Z}\otimes\mathrm{Z}),
\end{aligned}$$

and therefore,

$$\begin{aligned}
ccR_n(\theta) &= \left(\frac{7+e^{i\theta}}{8}\right)(\mathbb{I}\otimes\mathbb{I}\otimes\mathbb{I}) + \left(\frac{1-e^{i\theta}}{8}\right)\left((\mathbb{I}\otimes\mathbb{I}\otimes\mathrm{Z}) + (\mathbb{I}\otimes\mathrm{Z}\otimes\mathbb{I}) + (\mathrm{Z}\otimes\mathbb{I}\otimes\mathbb{I})\right) \\
&\quad - \left(\frac{1-e^{i\theta}}{8}\right)\left((\mathbb{I}\otimes\mathrm{Z}\otimes\mathrm{Z}) + (\mathrm{Z}\otimes\mathrm{Z}\otimes\mathbb{I}) + (\mathrm{Z}\otimes\mathbb{I}\otimes\mathrm{Z})\right) + \left(\frac{1-e^{i\theta}}{8}\right)(\mathrm{Z}\otimes\mathrm{Z}\otimes\mathrm{Z}).
\end{aligned}$$

And finally,

$$\begin{aligned}
ccR_z(\theta) &= \text{diag}\left(1,1,1,1,1,1,e^{-i\theta/2},e^{i\theta/2}\right) \\
&= \text{diag}\left(1,1,1,1,1,1,\cos\frac{\theta}{2},\cos\frac{\theta}{2}\right) + i\text{diag}\left(0,0,0,0,0,0,-\sin\frac{\theta}{2},\sin\frac{\theta}{2}\right) \\
&:= A + B.
\end{aligned}$$

We can expand A as,

$$\begin{aligned}
A &= \left(\frac{1+\cos\frac{\theta}{2}}{2}\right)(\mathbb{I}\otimes\mathbb{I}\otimes\mathbb{I}) + \left(\frac{1-\cos\frac{\theta}{2}}{2}\right)\text{diag}(1,1,1,1,1,1,-1,-1) \\
&= \left(\frac{1+\cos\frac{\theta}{2}}{2}\right)(\mathbb{I}\otimes\mathbb{I}\otimes\mathbb{I}) + \left(\frac{1-\cos\frac{\theta}{2}}{2}\right)(\text{CZ}\otimes\mathbb{I}),
\end{aligned} \tag{10}$$

and since from Equation 6,

$$\text{CZ}\otimes\mathbb{I} = \frac{1}{2}\left(\mathbb{I}\otimes\mathbb{I}\otimes\mathbb{I} + \text{Z}\otimes\mathbb{I}\otimes\mathbb{I} + \mathbb{I}\otimes\text{Z}\otimes\mathbb{I} - \text{Z}\otimes\text{Z}\otimes\mathbb{I}\right),$$

so we have

$$A = \left(\frac{3+\cos\frac{\theta}{2}}{4}\right)(\mathbb{I}\otimes\mathbb{I}\otimes\mathbb{I}) + \left(\frac{1-\cos\frac{\theta}{2}}{4}\right)(\text{Z}\otimes\mathbb{I}\otimes\mathbb{I} + \mathbb{I}\otimes\text{Z}\otimes\mathbb{I} - \text{Z}\otimes\text{Z}\otimes\mathbb{I}).$$

We can expand B as follows.

$$\begin{aligned}
B &= i\frac{\sin\frac{\theta}{2}}{4}(-\text{diag}(1,-1,1,-1,1,-1,1,-1) + \text{diag}(1,-1,-1,1,1,-1,-1,1) \\
&\quad +\text{diag}(1,-1,1,-1,-1,1,-1,1) - \text{diag}(1,-1,-1,1,-1,1,1,-1)) \\
&= i\frac{\sin\frac{\theta}{2}}{4}(-\mathbb{I}\otimes\mathbb{I}\otimes\text{Z} + \mathbb{I}\otimes\text{Z}\otimes\text{Z} + \text{Z}\otimes\mathbb{I}\otimes\text{Z} - \text{Z}\otimes\text{Z}\otimes\text{Z})
\end{aligned} \tag{11}$$

Therefore, we obtain the following.

$$\begin{aligned}
ccR_z(\theta) &= \left(\frac{3+\cos\frac{\theta}{2}}{4}\right)(\mathbb{I}\otimes\mathbb{I}\otimes\mathbb{I}) + \left(\frac{1-\cos\frac{\theta}{2}}{4}\right)(\text{Z}\otimes\mathbb{I}\otimes\mathbb{I} + \mathbb{I}\otimes\text{Z}\otimes\mathbb{I} - \text{Z}\otimes\text{Z}\otimes\mathbb{I}) \\
&\quad + i\frac{\sin\frac{\theta}{2}}{4}(-\mathbb{I}\otimes\mathbb{I}\otimes\text{Z} + \mathbb{I}\otimes\text{Z}\otimes\text{Z} + \text{Z}\otimes\mathbb{I}\otimes\text{Z} - \text{Z}\otimes\text{Z}\otimes\text{Z})
\end{aligned} \tag{12}$$

D Channel representation of $\widehat{G_{P_1,P_2}}$

Theorem D.1. *Let $G_{P_1,P_2} \in \mathcal{G}_{CS}$, where $P_1, P_2 \in \mathcal{P}_n \setminus \{\mathbb{I}\}$. Then its channel representation $\widehat{G_{P_1,P_2}}$ has the following properties.*

1. *The diagonal entries are 1 or $\frac{1}{2}$.*

2. *If a diagonal entry is 1 then all other entries in the corresponding row and column is 0.*

3. If a diagonal entry is $\frac{1}{2}$ then there exists three entries in the corresponding row and column that are equal to $\pm\frac{1}{2}$, rest is 0.

4. Exactly 2^{2n-2} i.e. $\frac{1}{4}^{th}$ of the diagonal elements are 1, while the remaining are $\frac{1}{2}$.

Proof. We recall that $G_{P_1,P_2} = \left(\frac{3+i}{4}\right)\mathbb{I}_n + \left(\frac{1-i}{4}\right)Q$, where $Q = P_1 + P_2 - P_1P_2$. The channel representation of a $2^n \times 2^n$ size unitary is a $2^{2n} \times 2^{2n}$ matrix, whose rows and columns are labeled by n-qubit Paulis. Let $P_r, P_s \in \mathcal{P}_n$. From definition, the $(P_r, P_s)^{th}$ element of $\widehat{G_{P_1,P_2}}$ is as follows.

$$\widehat{G_{P_1,P_2}}[P_r, P_s] = \frac{1}{2^n}\mathrm{Tr}\left(P_r G_{P_1,P_2} P_s G^\dagger_{P_1,P_2}\right) \tag{13}$$

Now,

$$\begin{aligned} P_r G_{P_1,P_2} P_s G^\dagger_{P_1,P_2} &= P_r\left[\left(\frac{3+i}{4}\right)\mathbb{I}_n + \left(\frac{1-i}{4}\right)Q\right]P_s\left[\left(\frac{3-i}{4}\right)\mathbb{I}_n + \left(\frac{1+i}{4}\right)Q\right] \\ &= \frac{5}{8}P_rP_s + \frac{1+2i}{8}P_rP_sQ + \frac{1-2i}{8}P_rQP_s + \frac{1}{8}P_rQP_sQ; \end{aligned} \tag{14}$$

and so using cyclic property of trace, from Equation 13 we have

$$\begin{aligned} \widehat{G_{P_1,P_2}}[P_r, P_s] &= \frac{5}{2^n \cdot 8}\mathrm{Tr}(P_rP_s) + \frac{1+2i}{2^n \cdot 8}\mathrm{Tr}(P_rP_sQ) + \frac{1-2i}{2^n \cdot 8}\mathrm{Tr}(P_sP_rQ) + \frac{1}{2^n \cdot 8}\mathrm{Tr}(P_rQP_sQ) \\ &:= a + b + c + d. \end{aligned} \tag{15}$$

Before proceeding, we observe that $\mathrm{Tr}[Q] = \mathrm{Tr}[P_1] + \mathrm{Tr}[P_2] - \mathrm{Tr}[P_1P_2] = 0$ since P_1, P_2 are distinct non-identity Paulis.

First row and column : Let us look at the first element i.e. $P_r = P_s = \mathbb{I}$. Then,

$$\begin{aligned} \widehat{G_{P_1,P_2}}[\mathbb{I}, \mathbb{I}] &= \frac{5}{2^n \cdot 8}\mathrm{Tr}[\mathbb{I}] + \frac{1+2i}{2^n \cdot 8}\mathrm{Tr}[Q] + \frac{1-2i}{2^n \cdot 8}\mathrm{Tr}[Q] + \frac{1}{2^n \cdot 8}\mathrm{Tr}[Q^2] \\ &= \frac{5}{8} + \frac{1}{2^n \cdot 8}\mathrm{Tr}[Q^2]. \end{aligned}$$

Now, since $[P_1, P_2] = 0$ we have

$$Q^2 = P_1^2 + P_2^2 + (P_1P_2)^2 + 2P_1P_2 - 2P_2P_1P_2 - 2P_1P_2P_1 = 3\mathbb{I} + 2P_1P_2 - 2P_1 - 2P_2, \tag{16}$$

and so $\widehat{G_{P_1,P_2}} = \frac{5}{8} + \frac{3}{8} = 1$.

Now let us consider any other element of the first row and column i.e. $P_r = \mathbb{I}, P_s \neq \mathbb{I}$.

$$\begin{aligned} a &= \frac{5}{2^n \cdot 8}\mathrm{Tr}[P_s] = 0 \\ b + c + d &= \frac{1+2i}{2^n \cdot 8}\mathrm{Tr}[P_sQ] + \frac{1-2i}{2^n \cdot 8}\mathrm{Tr}[P_sQ] + \frac{1}{2^n \cdot 8}\mathrm{Tr}[Q^2P_s] \\ &= \frac{1}{2^n \cdot 4}\mathrm{Tr}[P_sP_1 + P_sP_2 - P_sP_1P_2] + \frac{1}{2^n \cdot 8}\mathrm{Tr}[3P_s + 2P_1P_2P_s - 2P_1P_s - 2P_2P_s] \\ &= 0 \end{aligned}$$

So $\widehat{G_{P_1,P_2}}[\mathbb{I}, P_s] = 0$ and similarly $\widehat{G_{P_1P_2}}[P_r, \mathbb{I}] = 0$ where $P_r \neq \mathbb{I}$. Thus the first row and column has all 0, except the first entry where it is 1.

Diagonal i.e. $P_r = P_s$: Then we have

$$\begin{aligned} a &= \frac{5}{2^n\cdot 8}\mathrm{Tr}[P_r^2] = \frac{5}{8} \\ b &= \frac{1+2i}{2^n\cdot 8}\mathrm{Tr}[P_r^2 Q] = 0 \\ c &= \frac{1-2i}{2^n\cdot 8}\mathrm{Tr}[P_r^2 Q] = 0 \\ d &= \frac{1}{2^n\cdot 8}\mathrm{Tr}[(P_r Q)^2] \end{aligned}$$

Now

$$\begin{aligned} (P_r Q)^2 &= (P_r P_1 + P_r P_2 - P_r P_1 P_2)(P_r P_1 + P_r P_2 - P_r P_1 P_2) \\ &= (P_r P_1)^2 + P_r P_1 P_r P_2 - P_r P_1 P_r P_1 P_2 + P_r P_2 P_r P_1 + (P_r P_2)^2 - P_r P_2 P_r P_1 P_2 \\ &\quad - P_r P_1 P_2 P_r P_1 - P_r P_1 P_2 P_r P_2 + (P_r P_1 P_2)^2, \end{aligned} \tag{17}$$

and since $P_1 \neq P_2$ we have $\mathrm{Tr}[(P_r P_1)(P_r P_2)] = \mathrm{Tr}[(P_r P_1 P_2)(P_r P_2)] = \mathrm{Tr}[(P_r P_1 P_2)(P_r P_1)] = 0$, implying

$$d = \frac{1}{2^n\cdot 8}\left(\mathrm{Tr}[(P_r P_1)^2] + \mathrm{Tr}[(P_r P_2)^2] + \mathrm{Tr}[(P_r P_1 P_2)^2]\right).$$

If $[P_r, P_1] = [P_r, P_2] = 0$ then $d = \frac{3}{8}$ and $\widehat{G_{P_1,P_2}}[P_r, P_r] = 1$.

If $[P_r, P_1], [P_r, P_2] \neq 0$ then $[P_r, P_1, P_2] = 0$ and $d = -\frac{1}{8}$. If $[P_r, P_1] = 0$ but $[P_r, P_2] \neq 0$ then $[P_r, P_1 P_2] \neq 0$ and $d = -\frac{1}{8}$. Similarly, when $[P_r, P_2] = 0$ but $[P_r, P_1] \neq 0$ then $d = -\frac{1}{8}$. Thus, if P_r anti-commutes with at least one of P_1, P_2 we have $\widehat{G_{P_1,P_2}}[P_r, P_r] = \frac{5}{8} - \frac{1}{8} = \frac{1}{2}$. Thus the diagonal entries are either 1 or $\frac{1}{2}$.

Off-diagonal i.e. $P_r \neq P_s$: Then we have

$$\begin{aligned} a &= \frac{5}{2^n\cdot 8}\mathrm{Tr}(P_r P_s) = 0, \\ b + c &= \frac{1+2i}{2^n\cdot 8}\mathrm{Tr}(P_r P_s Q) + \frac{1-2i}{2^n\cdot 8}\mathrm{Tr}(P_s P_r Q), \\ \text{and } d &= \frac{1}{2^n\cdot 8}\mathrm{Tr}\left((P_r P_1 + P_r P_2 - P_r P_1 P_2) P_s Q\right) \\ &= \frac{1}{2^n\cdot 8}\left(\mathrm{Tr}(P_r P_1 P_s Q) + \mathrm{Tr}(P_r P_2 P_s Q) - \mathrm{Tr}(P_r P_1 P_2 P_s Q)\right) \\ &= \frac{1}{2^n\cdot 8}\left(\mathrm{Tr}(P_r P_1 P_s P_2) + \mathrm{Tr}(P_r P_2 P_s P_1) - \mathrm{Tr}(P_r P_1 P_s P_1 P_2)\right. \\ &\quad \left. - \mathrm{Tr}(P_r P_2 P_s P_2 P_1) - \mathrm{Tr}(P_r P_1 P_2 P_s P_1) - \mathrm{Tr}(P_r P_2 P_1 P_s P_2)\right), \end{aligned} \tag{18}$$

where the last line follows because $P_r \neq P_s$ and so $\mathrm{Tr}((P_r P_i)(P_s P_i)) = 0$. We consider the following cases.

Case I : $[\mathbf{P_r}, \mathbf{P_s}] = \mathbf{0}$: Then $b + c = \frac{1}{2^n\cdot 4}\left(\mathrm{Tr}(P_r P_s P_1) + \mathrm{Tr}(P_r P_s P_2) - \mathrm{Tr}(P_r P_s P_1 P_2)\right)$. Since $[P_r, P_s] = 0$ and $P_r \neq P_s$, so $P_r P_s = P' \in \pm\mathcal{P}_n \setminus \{\mathbb{I}\}$ (without imaginary phase). Now let us consider the following sub-cases.

Case Ia : $\mathbf{P_rP_s \neq \pm P_1, \pm P_2, \pm P_1P_2}$: In this case it is easy to prove that $b+c=0$. We can also prove that each summand in the expression of d is 0. For example, consider the term $\text{Tr}(P_rP_1P_sP_2)$, which can be non-zero if and only if $P_sP_2 = \pm(P_rP_1)$, implying $P_rP_s = \pm P_1P_2$. This is a contradiction to the assumptions in this case. Similar arguments hold for the other summands. Thus in this case $a+b+c+d=0$.

Case Ib : $\mathbf{P_rP_s = \pm P_1}$ or $\mathbf{\pm P_2}$ or $\mathbf{\pm P_1P_2}$: Let $P_sP_r = \pm P_1$ i.e. $P_r = \pm P_sP_1$. Then,

$$\begin{aligned}
b+c &= \pm\frac{1}{2^n\cdot 4}\text{Tr}\left((P_sP_1)^2\right) = \pm\frac{1}{4}, \\
d &= \pm\frac{1}{2^n\cdot 8}\left[\text{Tr}(P_sP_1P_1P_sQ) + \text{Tr}(P_sP_1P_2P_sQ) - \text{Tr}(P_sP_1P_1P_2P_sQ)\right] \\
&= \pm\frac{1}{2^n\cdot 8}\left[-\text{Tr}\left((P_sP_1P_2)^2\right) - \text{Tr}\left((P_sP_2)^2\right)\right]
\end{aligned}$$

Now here $[P_s,P_1]=0$. If $[P_s,P_2]=0$ then it implies $[P_s,P_1P_2]=0$. Then $d=\mp\frac{1}{4}$ and so $a+b+c+d=0$. On the other hand, if $[P_s,P_2]\neq 0$ then it implies $[P_s,P_1P_2]\neq 0$. Then $d=\pm\frac{1}{4}$ and so $a+b+c+d=\pm\frac{1}{2}$. Similar conclusions hold when $P_r=\pm P_sP_2$.

Let $P_r=\pm P_sP_1P_2$, which implies $[P_s,P_1P_2]=0$ i.e. P_s either commutes or anti-commutes with both P_1 and P_2.

$$\begin{aligned}
b+c &= \pm\frac{1}{2^n\cdot 4}\left(-\text{Tr}((P_sP_1P_2)^2)\right) = \mp\frac{1}{4} \\
d &= \pm\frac{1}{2^n\cdot 8}\left[\text{Tr}(P_sP_1P_2P_1P_sQ) + \text{Tr}(P_sP_1P_2P_2P_sQ) - \text{Tr}(P_sP_1P_2P_1P_2P_sQ)\right] \\
&= \pm\frac{1}{2^n\cdot 8}\left[\text{Tr}((P_sP_2)^2) + \text{Tr}((P_sP_1)^2)\right]
\end{aligned}$$

If $[P_s,P_1]=[P_s,P_2]=0$ then $d=\pm\frac{1}{4}$ and so $a+b+c+d=0$. If $[P_s,P_1],[P_s,P_2]\neq 0$ then $d=\mp\frac{1}{4}$ and so $a+b+c+d=\mp\frac{1}{2}$.

Case II : $\mathbf{[P_r,P_s]\neq 0}$: Then $b+c=\frac{i}{2^n\cdot 2}\left[\text{Tr}(P_rP_sP_1)+\text{Tr}(P_rP_sP_2)-\text{Tr}(P_rP_sP_1P_2)\right]$.

Case IIa : $\mathbf{P_rP_s \neq \pm iP_1, \pm iP_2, \pm iP_1P_2}$: We can prove $b+c=d=0$ with similar arguments as in Case Ia.

Case IIb : $\mathbf{P_rP_s = \pm iP_1}$ or $\mathbf{\pm iP_2}$ or $\mathbf{\pm iP_1P_2}$: Let $P_r=\pm iP_sP_1$, implying $[P_s,P_1]\neq 0$.

$$\begin{aligned}
b+c &= \frac{i}{2^n\cdot 2}\text{Tr}(\pm i(P_sP_1)^2) = \mp\frac{1}{2}\text{Tr}\left((P_sP_1)^2\right) = \pm\frac{1}{2} \\
d &= \pm\frac{i}{2^n\cdot 8}\left[\text{Tr}(P_sP_1P_1P_sQ) + \text{Tr}(P_sP_1P_2P_sQ) - \text{Tr}(P_sP_1P_1P_2P_sQ)\right] \\
&= \pm\frac{i}{2^n\cdot 8}\left[-\text{Tr}((P_sP_1P_2)^2) - \text{Tr}((P_sP_2)^2)\right]
\end{aligned}$$

If $[P_s,P_2]=0$ then $[P_s,P_1P_2]\neq 0$. If $[P_s,P_2]\neq 0$ then $[P_s,P_1P_2]=0$. Thus any one term in the above sum is +1 and the other is -1. So $d=0$ and $a+b+c+d=\pm\frac{1}{2}$. Similarly for $P_s=\pm iP_rP_2$.

Now consider $P_r=\pm iP_sP_1P_2$ i.e. $[P_s,P_1P_2]\neq 0$, implying P_s anti-commutes with either one of P_1 or P_2 but not both.

$$\begin{aligned}
b+c &= \pm\frac{i}{2^n\cdot 2}\text{Tr}(-i(P_sP_1P_2)^2) = \mp\frac{1}{2} \\
d &= \pm\frac{i}{2^n\cdot 8}\left[\text{Tr}(P_sP_1P_2P_1P_sQ) + \text{Tr}(P_sP_1P_2P_2P_sQ) - \text{Tr}(P_sP_1P_2P_1P_2P_sQ)\right] \\
&= \pm\frac{i}{2^n\cdot 8}\left[\text{Tr}((P_sP_2)^2) + \text{Tr}((P_sP_1)^2)\right]
\end{aligned}$$

If P_s anti-commutes with either P_1 or P_2, then one of the traces is 1 and the other is -1. So $d = 0$ and $b + c + d = \pm\frac{1}{2}$.

To summarize we have the following inferences.

1. $\widehat{G_{P_1,P_2}}[P_r, P_r] = 1$ when $[P_r, P_1] = [P_r, P_2] = 0$, else $\widehat{G_{P_1,P_2}}[P_r, P_r] = \frac{1}{2}$. This proves point (1) of the theorem. Point (4) of the theorem follows from the fact that two distinct Paulis can commute with exactly $\frac{1}{4}^{th}$ of the Paulis in $\mathcal{P}_n$.

2. From both Case I and II, we see that the constraints for $\widehat{G_{P_1,P_2}}[P_r, P_s] \neq 0$, implies P_r anti-commutes with one or both of P_1, P_2. This proves point (2).

3. Let P_r anti-commutes with one of P_1, P_2, say $[P_r, P_1] \neq 0$. Let $P_r P_1 = \pm i P_s \implies P_r = \pm i P_s P_1$. Then $\widehat{G_{P_1,P_2}}[P_r, P_s] = \pm\frac{1}{2}$ (Case II). This also implies that $[P_r, P_1 P_2] \neq 0$. Let $P_r P_1 P_2 = \pm i P'_s \implies P_r = \pm i P'_s P_1 P_2$. Then also $\widehat{G_{P_1,P_2}}[P_r, P'_s] = \mp\frac{1}{2}$ (Case II). Also, $[P_r, P_2] = 0$. Let $P_r P_2 = \pm P''_s \implies P_r = \pm P''_s P_2$. Then $\widehat{G_{P_1,P_2}}[P_r, P''_s] = \pm\frac{1}{2}$ (Case I). Thus there are $\pm\frac{1}{2}$ in 3 off-diagonal places, rest is 0.

   Let P_r anti-commutes with both P_1, P_2 i.e. $[P_r, P_1], [P_r, P_2] \neq 0$. Let $P_r P_1 = \pm i P_s \implies P_r = \pm i P_s P_1$ and $P_r P_2 = \pm i P'_s \implies P_r = \pm i P'_s P_2$. Then $\widehat{G_{P_1,P_2}}[P_r, P_s] = \widehat{G_{P_1,P_2}}[P_r, P'_s] = \pm\frac{1}{2}$ (Case II). This also implies that $[P_r, P_1 P_2] = 0$. Let $P_r P_1 P_2 = \pm P''_s \implies P_r = \pm P''_s P_1 P_2$ Then $\widehat{G_{P_1,P_2}}[P_r, P''_s] = \mp\frac{1}{2}$ (Case I). Here also, there are $\pm\frac{1}{2}$ in 3 off-diagonal places, rest is 0. This proves point (3) of the theorem.

□

D.1 Channel representation of $G^\dagger_{P_1,P_2}$

We know that G_{P_1,P_2} is a unitary and so its inverse is its adjoint i.e. $G^{-1}_{P_1,P_2} = G^\dagger_{P_1,P_2}$.

$$G^\dagger_{P_1,P_2} = \left(\frac{3+i}{4}\right)\mathbb{I} + \left(\frac{1-i}{4}\right)(P_1 + P_2 - P_1 P_2).$$

From Equation 14 we have,

$$\begin{aligned} P_r G^\dagger_{P_1,P_2} P_s G_{P_1,P_2} &= \left(P_r G_{P_1,P_2} P_s G^\dagger_{P_1,P_2}\right)^\dagger \\ &= \frac{5}{8} P_r P_s + \frac{1-2i}{8} P_r P_s Q + \frac{1+2i}{8} P_r Q P_s + \frac{1}{8} P_r Q P_s Q. \end{aligned}$$

Using cyclic property of trace we have

$$\begin{aligned} \widehat{G_{P_1,P_2}}[P_r, P_s] &= \frac{1}{2^n}\mathrm{Tr}\left(P_r G^\dagger_{P_1,P_2} P_s G_{P_1,P_2}\right) \\ &= \frac{5}{2^n \cdot 8}\mathrm{Tr}(P_r P_s) + \frac{1-2i}{2^n \cdot 8}\mathrm{Tr}(P_r P_s Q) + \frac{1+2i}{2^n \cdot 8}\mathrm{Tr}(P_s P_r Q) + \frac{1}{2^n \cdot 8}\mathrm{Tr}(P_r Q P_s Q) \\ &= a + \overline{b} + \overline{c} + d \end{aligned}$$

Whenever $b + c \in \mathbb{R}$, implying $\overline{b} + \overline{c} \in \mathbb{R}$, we have $\widehat{G_{P_1,P_2}}[P_r, P_s] = \widehat{G^\dagger_{P_1,P_2}}[P_r, P_s]$. This happens for the first row and column, diagonal entries, Case I of off-diagonal entries. So let us consider Case II, where $b + c \in \mathbb{C}$, without real parts, implying $\overline{b} + \overline{c} = -(b + c)$. We have seen that in Case II $a = d = 0$ and the contribution to non-zero value comes only from $b + c$. So at those off-diagonal entries we have $\widehat{G_{P_1,P_2}}[P_r, P_s] = -\widehat{G^\dagger_{P_1,P_2}}[P_r, P_s]$ - the sign flips.

E Pseudocode

Here we give the pseudocodes and brief explanations for the algorithms described in Sections 3, 4 and 5. We have not included the pseudocodes for some small sub-routines. We preferred to simply describe briefly about them in relevant places. We denote $N = 2^n$, where n is the number of qubits.

I. Generating set $\mathcal{G}_{\mathbf{CS}}$: Algorithm 1 (**GEN-CS**) takes as input n, the number of qubits and outputs the generating set $\mathcal{G}_{CS}$ as an array of pairs of Paulis that represent the unitaries in this set, satisfying some properties given by Lemma 3.1, as described in Section 3. It calls the sub-routine **PAULI-COMM** that tests if two n-qubit Paulis commute. This can be done by checking if there are even number of qubit-wise anti-commutations.

II. Channel representation : In Algorithm 2 (**CHAN-CS**) we have given the pseudocode for computing the channel representation of each element in $\mathcal{G}_{CS}$. $\widehat{G_{P_1,P_2}}$ is stored as an array A_{P_1,P_2} of size $3 \cdot 2^{2n-2}$, as discussed in Section 4. Each element of this array is of the form $[i, \pm j_1, \pm j_2, \pm j_3]$, implying $\widehat{G_{P_1,P_2}}[i,i] = \frac{1}{2}$, $\widehat{G_{P_1,P_2}}[i,j_1] = \pm\frac{1}{2}$, $\widehat{G_{P_1,P_2}}[i,j_2] = \pm\frac{1}{2}$ and $\widehat{G_{P_1,P_2}}[i,j_3] = \pm\frac{1}{2}$.

Alternatively, we can compute the channel representation using the commutation relations, as shown in the proof of Theorem D.1. For example, $\widehat{G_{P_1,P_2}}[P_r,P_r] = 1$ if $P_r, P_1 = [P_r, P_2] = 0$, else it is $\frac{1}{2}$. For the latter, the remaining 3 off-diagonal elements can be determined using the product relations, as shown in the proof of the above-mentioned theorem.

III. Multiplication by $\widehat{\mathbf{G_{P_1,P_2}}}$: The pseudocode for efficiently multiplying a unitary with $\widehat{G_{P_1,P_2}}$ has been given in Algorithm 5 (**MULT-$\mathcal{G}_{\mathbf{CS}}$**). More detail explanations have been provided in Section 4.1. We keep in mind that this algorithm is used while working with exactly implementable unitaries, whose channel representation are matrices with elements in $\mathbb{Z}\left[\frac{1}{2}\right]$ and hence are represented as $[a,k]$, implying $\frac{a}{2^k}$. It calls the sub-routine **ADD-2** (Algorithm 4), which adds two elements in $\mathbb{Z}\left[\frac{1}{2}\right]$, where each element $v = \frac{a}{2^k}$ is represented as $[a,k]$. This in turn calls Algorithm 3 (**$\mathbf{sde_2}$-REDUCE**), which reduces a fraction $v = \frac{a}{2^k}$ to $\frac{a'}{2^{k'}}$ such that $k' = \text{sde}_2(v)$.

III. Approximately implementable unitaries : The pseudocodes for the implementation of the algorithm to find optimal CS-count of approximately implementable unitaries, as outlined in Section 5.1, are as follows.

The optimization version, **APPROX-CS-OPT** (Algorithm 6),takes as input a unitary $W \in \mathcal{U}_n$ and precision or error parameter $\epsilon > 0$. It iteratively calls the decision version, **APPROX-CS-DECIDE** (Algorithm 7), that has an additional parameter $m \in \mathbb{Z}_{>0}$ and outputs YES if there exists an exactly implementable unitary U within distance ϵ of W and with CS-count at most m. It loops over all products of m unitaries from $\mathcal{G}_{CS}$ and in each iteration it calculates a set of amplitudes (step 4) in order to perform the amplitude test (step 8), as described in Section 5.1. If it passes this test then it performs a conjugation test $\mathcal{A}_{\mathbf{CONJ}}$ (Algorithm 8). It decides if an input unitary W' is "close to a Clifford" i.e. $W' = EC_0$, where $C_0 \in \mathcal{C}_n, E \in \mathcal{U}_n$ and $d(E, \mathbb{I}) \leq \epsilon$. APPROX-CS-DECIDE returns a YES if it passes both the tests.

V. Nested meet-in-the-middle : We have given the pseudocode for the nested meet-in-the-middle (MITM) algorithm described in Section 5.2.1 in Algorithm 9 (**Nested MITM**).

VI. Exactly implementable unitaries : The pseudocodes for the implementation of the heuristic algorithm to find a CS-count-optimal decomposition of exactly implementable unitaries, as explained in Section 5.2.2, are as follows.

The optimization version, **EXACT-CS-OPT** (Algorithm 10) iteratively calls the decision version, **EXACT-CS-DECIDE** (Algorithm 11). Since the sde_2 of a unitary can change by at most 1 after multiplication by $\widehat{G_{P_1,P_2}}$ (Lemma 4.10), so we start testing from $\text{sde}_2(\widehat{U})$, where U is the input unitary.

Algorithm 11 tests if the CS-count of an input unitary is at most a certain integer m, and if so it returns a decomposition. The procedure is described in Section 5.2.2, so we give a brief explanation here. In short, it builds a pruned tree, where the input unitary is the root and a Clifford is a leaf. Each edge is multiplication by a generating set element (step 8). We use a 3×3 integer matrix SH, whose rows index sde increase, unchanged and decrease. The columns index hamming weight increase, unchanged and decrease. This matrix is used in order to divide the children nodes according to their change in sde and hamming weight with respect to the parent node (step 12). Then we select a subset of the children nodes which either belong to the minimum cardinality set from the SH matrix (steps 19, 25) or have sde 1 (step 28). If sde of any node is 0 (implying a Clifford or leaf) then we return the decomposition (step 10).

We use a number of sub-routines. **HAM-WT-MAT** finds the Hamming weight of an input unitary. **UPDATE-SH** updates the SH matrix according to the Divide-and-Select rule used. **MIN-SH** returns the row and column index of the minimum non-zero entry of the SH matrix.

VII. Random channel representation : In Algorithm 12 (**RANDOM-CHAN-REP**) we generate the channel representation of a random unitary whose CS-count is at most some input integer cs_{in}. First cs_{in} number of unitaries are randomly selected from $\widehat{\mathcal{G}_{CS}}$ and multiplied. Then we randomly permute the columns of the resultant unitary. After that we multiply each column by -1 with probability 1/2.

References

[1] David Gosset, Vadym Kliuchnikov, Michele Mosca, and Vincent Russo. An algorithm for the T-count. *Quantum Information & Computation*, 14(15-16):1261–1276, 2014.

[2] Vlad Gheorghiu, Michele Mosca, and Priyanka Mukhopadhyay. T-count and T-depth of any multi-qubit unitary. *npj Quantum Information*, 8(1):141, 2022.

[3] Salman Beigi and Peter W Shor. C_3, semi-Clifford and generalized semi-Clifford operations. *Quantum Information and Computation*, 10(1-2):41–59, 2010.

Algorithm 1: GEN-CS

Input: n=number of qubits.

Output: Generating set $\mathcal{G}_{CS}$ as set of pairs of Paulis that represent the unitaries in this set.

```
G_CS = [] ;
for each P_1 ∈ P_n \ {I} do
    for each P_2 ∈ P_n \ {I} and P_2 > P_1 do
        if PAULI-COMM(P_1, P_2)==YES then
            flag = 1 ;
            for each (P_a, P_b) ∈ G_CS do
                prod = P_a P_b; match = 0 ;
                R = (P_a, P_b); R' = (P_1, P_2); Q = (I, I); indx_1 = (0, 1); indx_2 = (0, 1) ;
                for j = 0, 1 do
                    for k = 0, 1 do
                        if R[j] == R'[k] then
                            Q[j] = R'[k]; match = 1; indx_1 = indx_1 \ {j}; indx_2 = indx_2 \ {k} ;
                        end
                    end
                end
                if match == 1 then
                    Q[indx_1[0]] = R'[indx_2[0]] ;
                    if prod == ±Q[0] or ±Q[1] then
                        flag = 0 ;
                        break ;
                    end
                end
            end
            if flag == 1 then
                G_CS.append(P_1, P_2) ;
            end
        end
    end
end
return G_CS ;
```

Algorithm 2: CHAN-CS

Input: (i) n = number of qubits; (ii) $\mathcal{G}_{CS}$

Output: $\widehat{\mathcal{G}_{CS}} = \{A_{P_1,P_2} : P_1, P_2 \in \mathcal{P}_n; \quad \text{array } A_{P_1,P_2} \text{ represents } \widehat{G_{P_1,P_2}}\}$.

$\widehat{\mathcal{G}_{CS}} = []$;
for $(P_1, P_2) \in \mathcal{G}_{CS}$ **do**
    $A_{P_1,P_2} = []; \qquad Q = P_1 + P_2 - P_1 P_2$;
    for $P_r \in \mathcal{P}_n$ **do**
        $tuple = []$;
        $val = \frac{1}{2^n}\left(\frac{5}{8}\mathrm{Tr}(\mathbb{I}) + \frac{1}{8}\mathrm{Tr}((P_r Q)^2)\right)$;
        if $val == \frac{1}{2}$ **then**
            $tuple.\mathrm{append}(P_r); \qquad num = 0$;
            for $P_s \in \mathcal{P}_n \setminus \{P_r\}$ **do**
                $val = \frac{1}{2^n}\left(\frac{1+2i}{8}\mathrm{Tr}(P_r P_s Q) + \frac{1-2i}{8}\mathrm{Tr}(P_s P_r Q) + \frac{1}{8}\mathrm{Tr}(P_r Q P_s Q)\right)$;
                if $val == \pm\frac{1}{2}$ **then**
                    $tuple.\mathrm{append}(\pm P_s); \qquad num = num + 1$;
                    if $num == 3$ **then**
                        $A_{P_1,P_2}.\mathrm{append}(tuple)$;
                        break ;
                    end
                end
            end
        end
    end
    $\widehat{\mathcal{G}_{CS}}.\mathrm{append}(A_{P_1,P_2})$;
end
return $\widehat{\mathcal{G}_{CS}}$;

Algorithm 3: sde_2-REDUCE

Input: $v = (a, k) \in \mathbb{Z}\left[\frac{1}{2}\right]$.

Output: $v = (a', k')$ such that $k' = \mathrm{sde}_2(v)$.

while $a\%2 == 0$ *and* $k! = 0$ **do**
    $a \leftarrow a/2$;
    $k \leftarrow k - 1$;
end
return (a, k) ;

Algorithm 4: ADD-2

Input: $v_1 = (a_1, k_1)$, $v_2 = (a_2, k_2) \in \mathbb{Z}\left[\frac{1}{2}\right]$.

Output: $v = (a, k) = v_1 + v_2$

if $k_1 \geq k_2$ **then**
    $num = a_1 + a_2 \cdot 2^{k_1 - k_2}$;
    $den = k_1$;
else
    $num = a_1 \cdot 2^{k_2 - k_1} + a_2$;
    $den = k_2$;
end
return sde_2-REDUCE $((num, den))$;

Algorithm 5: MULT-$\mathcal{G}_{CS}$

Input: (i) $\widehat{G_{P_1P_2}}$ as array A_{P_1,P_2}, (ii) U - both of size $N^2 \times N^2$.
Output: (i) $U_p = \widehat{G_{P_1P_2}}U$.

for $i = 1, \ldots, \frac{3N^2}{4}$ **do**
  $diag = A_{P_1,P_2}[i][0]$; $oDiag_1 = A_{P_1,P_2}[i][1]$; $oDiag_2 = A_{P_1,P_2}[i][2]$; $oDiag_3 = A_{P_1,P_2}[i][3]$;
  if $oDiag_1 < 0$ **then**
    $oDiagIndx_1 = -oDiag_1$;
  else
    $oDiagIndx_1 = oDiag_1$;
  end
  if $oDiag_2 < 0$ **then**
    $oDiagIndx_2 = -oDiag_2$;
  else
    $oDiagIndx_2 = oDiag_2$;
  end
  if $oDiag_3 < 0$ **then**
    $oDiagIndx_3 = -oDiag_3$;
  else
    $oDiagIndx_3 = oDiag_3$;
  end
  for $j = 1, \ldots, N^2$ **do**
    $v_1 = (U[diag, j][0], U[diag, j][1] + 1)$;
    if $oDiag_1 < 0$ **then**
      $v_2 = (-U[oDiagIndx_1, j][0], U[oDiagIndx_1, j][1] + 1)$;
    else
      $v_2 = (U[oDiagIndx_1, j][0], U[oDiagIndx_1, j][1] + 1)$;
    end
    if $oDiag_2 < 0$ **then**
      $v_3 = (-U[oDiagIndx_2, j][0], U[oDiagIndx_2, j][1] + 1)$;
    else
      $v_3 = (U[oDiagIndx_2, j][0], U[oDiagIndx_2, j][1] + 1)$;
    end
    if $oDiag_3 < 0$ **then**
      $v_4 = (-U[oDiagIndx_3, j][0], U[oDiagIndx_3, j][1] + 1)$;
    else
      $v_4 = (U[oDiagIndx_3, j][0], U[oDiagIndx_3, j][1] + 1)$;
    end
    $U_p[diag, j] =$ ADD-2(ADD-2(v_1, v_2), ADD-2(v_3, v_4)) ;
  end
end
return U_p ;

Algorithm 6: APPROX-CS-OPT

Input: (i) $W \in \mathcal{U}_n$, (ii) $\epsilon \geq 0$.
Output: $\mathcal{S}_\epsilon(W)$.

$m \leftarrow 1$, *decision* $\leftarrow$ NO ;
while *(1)* **do**
  decision $\leftarrow$ APPROX-CS-DECIDE (W, m, ϵ) ;
  if *decision* == *YES* **then**
    return m ;
  else
    $m \leftarrow m + 1$;
  end
end

Algorithm 7: APPROX-CS-DECIDE

Input: (i) $W \in \mathcal{U}_n$, (ii) integer $m > 0$, (iii) $\epsilon \geq 0$.
Output: YES if $\exists U \in \mathcal{J}_n^{CS}$ such that $d(U,W) \leq \epsilon$ and $\mathcal{S}(U) \leq m$; else NO.

$\mathcal{G}_{CS} \leftarrow$ GEN-CS(n) ;
for *every* $\widetilde{U} = \prod_{i=m}^{1} G_{P_{1j}P_{2j}}$ *such that* $G_{P_{1j}P_{2j}} \in \mathcal{G}_{CS}$ *and* $(P_{1j}, P_{2j}) \neq (P_{1,j+1}, P_{2,j+1})$ **do**
  $W' = W^{\dagger}\widetilde{U}$;
  $A_c \leftarrow \{|\mathrm{Tr}\left(W'P\right)/N| : P \in \mathcal{P}_n\}$ and sort this set in descending order ;
  for $M = 1, 2 \ldots N^2$ **do**
    $A_1 \leftarrow$ First M terms in A_c ;
    $A_0 = A_c \setminus A_1$;
    if *each term in* $A_1 \in \left[\frac{1-\epsilon^2}{\sqrt{M}} - \sqrt{M(2\epsilon^2-\epsilon^4)}, \frac{1}{\sqrt{M}} + \sqrt{M(2\epsilon^2-\epsilon^4)}\right]$ *and each term in* $A_0 \in \left[0, \sqrt{M(2\epsilon^2-\epsilon^4)}\right]$ **then**
      if *YES* $\leftarrow \mathcal{A}_{CONJ}(W', \epsilon)$ **then**
        return YES ;
      end
    end
  end
end
return NO;

Algorithm 8: $\mathcal{A}_{CONJ}$

Input: (i) $W' \in \mathcal{U}_n$, (ii) $\epsilon \geq 0$.
Output: YES if $\exists C_0 \in \mathcal{C}_n, E \in \mathcal{U}_n$ such that $W' = E^{\dagger}C_0$, where $d(E, \mathbb{I}) \leq \epsilon$; else NO.

$p \leftarrow 1$;
for *every* $P_{out} \in \mathcal{P}_n$ **do**
  if $p == 1$ **then**
    $p \leftarrow 0$;
  end
  for *every* $P_{in} \in \mathcal{P}_n$ **do**
    if $(1 - 4\epsilon^2 + 2\epsilon^4) \leq \left|Tr\left(W'P_{out}W'^{\dagger}P_{in}\right)\right|/N \leq 1$ **then**
      $p \leftarrow p + 1$;
      if $p > 1$ **then**
        return NO ;
      end
    end
    if $2\epsilon < \left|Tr\left(W'P_{out}W'^{\dagger}P_{in}\right)\right|/N < (1 - 4\epsilon^2 + 2\epsilon^4)$ **then**
      return NO ;
    end
  end
end
return YES ;

Algorithm 9: Nested MITM

Input: (i) A unitary $U \in \mathcal{J}_n^{CS}$, (ii) generating set $\mathcal{G}_{CS}$, (iii) test-count m, (iv) $c \geq 2$

Output: A circuit (if it exists) for U such that CS-count is at most m.

$S_0 \leftarrow \{\mathbb{I}\}$; $i \leftarrow 1$;
while $i \leq \lceil \frac{m}{c} \rceil$ **do**
  $S_i \leftarrow \{\left(\widehat{G_{P_1P_2}}\widehat{W}\right)^{(co)} : G_{P_1P_2} \in \mathcal{G}_{CS}, W \in S_{i-1}\}$; $k = c - 1$;
  for $\widehat{W} = \widehat{W_1}\widehat{W_2}\ldots\widehat{W_k}$ *where* $\widehat{W_i} \in S_i$ *or* $\widehat{W_i} \in S_{i-1}$ **do**
    if $\exists \widehat{W'} \in S_i$ *such that* $\left(\widehat{W}^\dagger \widehat{U}\right)^{(co)} = \widehat{W'}$ **then**
      return $\widehat{W_1}, \widehat{W_2}, \ldots, \widehat{W_k}, \widehat{W'}$;
      break ;
    end
    else if $\exists \widehat{W'} \in S_{i-1}$ *such that* $\left(\widehat{W}^\dagger \widehat{U}\right)^{(co)} = \widehat{W'}$ **then**
      return $\widehat{W_1}, \widehat{W_2}, \ldots, \widehat{W_k}, \widehat{W'}$;
      break ;
    end
  end
  $i \leftarrow i + 1$;
end
if *no decomposition found* **then**
  return "U has CS-count more than m." ;
end

Algorithm 10: EXACT-CS-OPT

Input: $\widehat{U}$, where $U \in \mathcal{J}_n^{CS}$

Output: m, the minimum CS-count of U and its decomposition $\widehat{U} = \left(\prod_{j=m}^{1} \widehat{G_{P_{1j}P_{2j}}}\right)\widehat{C_0}$, where $G_{P_{1j}P_{2j}} \in \mathcal{G}_{CS}$.

$m = \text{sde}_{\widehat{U}}$;
if $m == 0$ **then**
  return 0 ;
else
  while *1* **do**
    $(m', \mathcal{D}) \leftarrow$ EXACT-CS-DECIDE$(\widehat{U}, m)$;
    if $m' == -1$ **then**
      $m \leftarrow m + 1$;
    else
      return $(m', \mathcal{D})$;
    end
  end
end

Algorithm 11: EXACT-CS-DECIDE

Input: (i)$\widehat{U}$ where $U \in \mathcal{J}_n^{CS}$; (ii) $m \in \mathbb{Z}_{>0}$; (iii) $\widehat{\mathcal{G}_{CS}}$.

Output: $(m', \mathcal{D})$, where $m' \in \mathbb{Z}$ is -1 if CS-count is more than m and $\mathcal{D}$ is a decomposition if CS-count is $m' \geq 1$.

$\widetilde{U} = []$; $\quad parNode = [\widehat{U}, \text{Path}_{\widehat{U}}, \text{sde}_{\widehat{U}}, \text{ham}_{\widehat{U}}]$; $\quad parNode = \{\widetilde{U}\}$ `// Root node` ;
for $i = 1, \ldots, m$ **do**
- $childNode = []$; $\quad SH = [0]_{3\times 3}$;
- **for** *each* $\widetilde{U} = [\widehat{U}, Path_{\widehat{U}}, sde_{\widehat{U}}, ham_{\widehat{U}}] \in parNode$ **do**
  - $P_{prev} = \text{Path}_{\widehat{U}}[i-1]$; $\quad \text{sde}_{par} = \text{sde}_{\widehat{U}}$; $\quad \text{ham}_{par} = \text{ham}_{\widehat{U}}$;
  - **for** *each* $\widehat{G_{P_1,P_2}} \in \widehat{\mathcal{G}_{CS}}$ **do**
    - **if** $(P_1, P_2) \neq P_{prev}$ **then**
      - $W \leftarrow \text{MULT-}\mathcal{G}_{CS}(\widehat{G_{P_1,P_2}}, \widehat{U})$; $\quad \text{ham}_W \leftarrow \text{HAM-WT-MAT}(W)$; $\quad \text{sde}_W \leftarrow \text{GET-SDE}(W)$;
      - **if** $sde_W == 0$ **then**
        - **return** $(i, \text{Path}_{\widehat{U}} \cup \{(P_1, P_2)\})$ `// Reached Clifford` ;
      - **else**
        - $(SH, s, h) \leftarrow$UPDATE-SH $(SH, \text{sde}_W, \text{ham}_W, \text{sde}_{par}, \text{ham}_{par}, rule)$;
      - **end**
      - $\text{Path}_W = \text{Path}_{\widehat{U}} \cup \{(P_1, P_2)\}$;
      - $childNode$.append$(W, s, h, \text{Path}_W, \text{sde}_W, \text{ham}_W)$;
    - **end**
  - **end**
- **end**
- $(s_{indx}, h_{indx}) \leftarrow$ MIN-SH(SH) ;
- **for** *each* $\widetilde{W} = [W, s, h, Path_W, sde_W, ham_W] \in childNode$ **do**
  - **if** $sde_W > m + 1 - i$ **then**
    - **continue** ;
  - **end**
  - **if** $sde_W == 1$ **then**
    - $parNode$.append$([W, \text{Path}_W, \text{sde}_W, \text{ham}_W])$;
  - **end**
  - **if** $s == s_{indx}$ *and* $h == h_{indx}$ **then**
    - $parNode$.append$([W, \text{Path}_W, \text{sde}_W, \text{ham}_W])$;
  - **end**
- **end**

end
return $(-1, [])$;

Algorithm 12: RANDOM-CHAN-REP

Input: cs_{in} : Input CS-count

Output: $\widehat{U}$: Channel representation of a unitary with CS-count at most cs_{in}

$i = 0$; $\quad \widehat{U} = \widehat{\mathbb{I}}$;
while $i < cs_{in}$ **do**
- Randomly sample (P_1, P_2) from $\mathcal{G}_{CS}$ `// As output by GEN-CS (Algorithm 1 )`;
- **if** $i == 0$ **then**
  - $P_{prev} = (P_1, P_2)$;
  - $\widehat{U} \leftarrow \text{MULT-}\mathcal{G}_{CS}(\widehat{G_{P_1,P_2}}, \widehat{U})$;
- **else**
  - **if** $(P_1, P_2) \neq P_{prev}$ **then**
    - $P_{prev} = (P_1, P_2)$;
    - $\widehat{U} \leftarrow \text{MULT-}\mathcal{G}_{CS}(\widehat{G_{P_1,P_2}}, \widehat{U})$;
  - **end**
- **end**

end
Randomly permute the columns of $\widehat{U}$;
Multiply each column of $\widehat{U}$ with -1 with probability $\frac{1}{2}$;
return $\widehat{U}$;
